# Decoding the enigma: unveiling the transmission characteristics of waterfowl-associated *bla*_NDM-5_-positive *Escherichia coli* in select regions of China

**DOI:** 10.3389/fmicb.2024.1501594

**Published:** 2024-12-09

**Authors:** Shaqiu Zhang, Yanxi Shu, Zhechen Yang, Zhijun Zhong, Mingshu Wang, Renyong Jia, Shun Chen, Mafeng Liu, Dekang Zhu, Xinxin Zhao, Ying Wu, Qiao Yang, Juan Huang, Xumin Ou, Di Sun, Bin Tian, Zhen Wu, Yu He, Anchun Cheng

**Affiliations:** ^1^Avian Disease Research Center, College of Veterinary Medicine, Sichuan Agricultural University, Chengdu, China; ^2^Institute of Veterinary Medicine and Immunology, Sichuan Agricultural University, Chengdu, China; ^3^Key Laboratory of Animal Disease and Human Health of Sichuan Province, Sichuan Agricultural University, Chengdu, China; ^4^Engineering Research Center of Southwest Animal Disease Prevention and Control Technology, Ministry of Education of the P.R. China, Chengdu, China

**Keywords:** *bla*_NDM-5_, *Escherichia coli*, antibiotic resistance, horizontal gene transfer, plasmid

## Abstract

*Escherichia coli* (*E. coli*) serves as a critical indicator microorganism for assessing the prevalence and dissemination of antibiotic resistance, notably harboring various antibiotic-resistant genes (ARGs). Among these, the emergence of the *bla*_NDM_ gene represents a significant threat to public health, especially since carbapenem antibiotics are vital for treating severe infections caused by Gram-negative bacteria. This study aimed to characterize the antibiotic resistance features of *bla*_NDM-5_-positive *E. coli* strains isolated from waterfowl in several regions of China and elucidate the dissemination patterns of the *bla*_NDM-5_ gene. We successfully isolated 103 *bla*_NDM-5_-positive *E. coli* strains from 431 intestinal fecal samples obtained from waterfowl across five provincial-level units in China, with all strains exhibiting multidrug resistance (MDR). Notably, the *bla*_NDM-5_ gene was identified on plasmids, which facilitate efficient and stable horizontal gene transfer (HGT). Our adaptability assays indicated that while the *bla*_NDM-5_-positive plasmid imposed a fitness cost on the host bacteria, the NDM-5 protein was successfully induced and purified, exhibiting significant enzymatic activity. One strain, designated DY51, exhibited a minimum inhibitory concentration (MIC) for imipenem of 4 mg/L, which escalated to 512 mg/L following exposure to increasing imipenem doses. This altered strain demonstrated stable resistance to imipenem alongside improved adaptability, correlating with elevated relative expression levels of the *bla*_NDM-5_ and overexpression of efflux pumps. Collectively, this study highlights the horizontal dissemination of the *bla*_NDM-5_ plasmid among *E. coli* strains, confirms the associated fitness costs, and provides insights into the mechanisms underlying the stable increase in antibiotic resistance to imipenem. These findings offer a theoretical framework for understanding the dissemination dynamics of *bla*_NDM-5_ in *E. coli*, which is essential for developing effective strategies to combat carbapenem antibiotic resistance.

## Introduction

The discovery and use of antimicrobial agents have significantly advanced human public health, animal welfare, and agriculture (Endale et al., 2023). However, the rapid growth of antimicrobial resistance (AMR) and the emergence of “superbugs” pose significant challenges to global public health (Mitra et al., 2022; Collineau et al., 2023). In intensive farming operations, antimicrobial agents are often employed to prevent and treat animal diseases, which can lead to the presence of residual antibiotics in the environment (Wang X. R. et al., 2021; Tang et al., 2022). Consequently, antibiotic-resistant bacteria frequently withstand the pressure of antimicrobial agents through their acquired ARGs, which are capable of HGT (Christaki et al., 2020). *Escherichia coli* is a common Gram-negative bacterium, typically found as a symbiotic inhabitant in the intestines of humans and animals, and it can be frequently detected in the environment (Arbab et al., 2022). Pathogenic strains of *E. coli* can cause various diseases, such as urinary tract infections, bacteremia, cystitis, and others (Croxen et al., 2013). As a result, *E. coli* is often used as an indicator for monitoring antibiotic resistance and is also regarded as a reservoir for ARGs, which can be horizontally transferred to other bacteria, posing a serious threat to public health and safety (Zhang et al., 2020; Zhang et al., 2021a; Li Q. et al., 2024).

Compared to other antibiotics, carbapenem antibiotics exhibit excellent antibacterial activity, a broad spectrum of coverage, and enhanced stability against *β*-lactamases. They are commonly used to treat severe bacterial infections and are thus considered “a class of last-resort antibiotics” (Ma et al., 2023). However, the frequent use of carbapenem antibiotics has led to an increase in infections caused by carbapenem-resistant *Enterobacterales* (CRE), which has become one of the three most significant challenges in the global fight against infections. Although carbapenem antibiotics are banned for use in animals in China, the incidence of CRE isolated from animal sources continues to rise (Poirel et al., 2018).

Since the discovery of the first patient carrying the *bla*_NDM-1_ gene in India in 2008 (Yong et al., 2009), 41 variants of *bla*_NDM_ have been identified. Currently, the most prevalent types are *bla*_NDM-1_ and *bla*_NDM-5_; notably, *bla*_NDM-5_ is commonly found in animals in China. Various studies have detected *bla*_NDM-5_-positive *E. coli* in livestock, poultry, and the surrounding environment of farms in China (He et al., 2017; Wang M. G. et al., 2021; He et al., 2023). It is evident that *bla*_NDM-5_-producing CRE are widespread in China, particularly among livestock and poultry, where *bla*_NDM-5_ appears to be the predominant variant. At present, carbapenem antibiotics are approved solely for clinical use, but the presence of *bla*_NDM_ has been observed in animals, the environment, and humans. The spread of *bla*_NDM-5_ is primarily facilitated by plasmids, which play a crucial role in the evolution of bacterial antibiotic resistance. Plasmids contribute to the spread of ARGs through HGT, creating conditions conducive to the emergence of “superbugs” (Lerminiaux and Cameron, 2019; Das, 2023; Pu et al., 2023).

Frequent or inappropriate use of antibiotics often prompts bacteria to develop resistance, which can increase the risk of MDR bacteria (Chen et al., 2024; Guan et al., 2024). One of the most evident changes is the alteration of the MIC value. In addition, bacteria may exhibit multidrug resistance and cross-resistance (Gu et al., 2020; Li et al., 2020; Li et al., 2022), further enhancing their survival capabilities in antibiotic-rich environments. These changes are coping strategies employed by bacteria in response to environmental pressures and are of significant importance in studying the mechanisms of bacterial resistance.

In this study, 103 MDR *bla*_NDM-5_-positive *E. coli* strains were isolated and identified from waterfowl source samples in select regions of China. Furthermore, one *bla*_NDM-5_-positive *E. coli* strain (DY51) was observed to be inducible from low-level resistance (MIC = 4 mg/L) to high-level resistance (MIC = 512 mg/L). Studying the molecular transmission characteristics of *bla*_NDM-5_ can enhance our understanding of its transmission pathways in the environment and its implications for animals and humans, laying a foundation for further research on the specific resistance mechanisms of *bla*_NDM-5_. This study not only deepens our understanding of carbapenem antibiotic resistance in *bla*_NDM-5_-positive *E. coli* strains derived from waterfowl but also provides new insights and methods for preventing, controlling, and treating antibiotic resistance.

## Materials and methods

### Study design, isolation, and identification of positive *bla*_NDM-5_ *Escherichia coli*

China is a major producer of waterfowl. To investigate the prevalence of *bla*_NDM-5_ carried by bacteria in waterfowl, our research team established close contact with numerous domestic farms from 2021 to 2023. This collaboration enabled us to collect a significant number of disease samples from the Sichuan, Shaanxi, Chongqing, Xinjiang, and Anhui regions, which are provincial-level administrative units in China. A total of 431 carcasses of diseased ducks were sent by farmers to the laboratory under cold conditions for testing. We obtained intestinal fecal contents by dissecting the carcasses in a sterile laboratory to ensure that the isolated bacteria accurately represent the local situation without contamination from various environmental factors. The intestinal content samples were initially inoculated in Luria-Bertani (LB) broth (HOPEBIO, Qingdao, China, #HB0128) at 37°C for enrichment culture, followed by inoculation onto MacConkey agar (HOPEBIO, Qingdao, China, #HB6238) containing 4 mg/L of imipenem (Meilun Biotechnology Co., Ltd., Dalian). Pink colonies were subjected to further identification using 16S rRNA sequencing with universal primers. The identified *E. coli* isolates were preserved in LB broth supplemented with 30% glycerol and stored at −80°C for subsequent analyses. Imipenem-resistant strains were selected and subjected to PCR amplification and sequencing. Universal 16S rRNA and *bla*_NDM-5_ identification primers were used to identify *bla*_NDM-5_-positive *E. coli* (Hornsey et al., 2011; Zhang et al., 2021b).

### Carbapenemase phenotype screening

The modified Hodge test (MHT), modified carbapenem inactivation test (mCIM), and EDTA-modified carbapenem inactivation test (eCIM) for all *bla*_NDM-5_-positive *E. coli* isolates were performed according to the standards of the American Clinical and Laboratory Standards Institute (CLSI) (Humphries et al., 2021). Isolates were resuspended in 1.5 mL of LB and then treated with 2 mL of LB containing 5 mM EDTA (15 μL of 0.5 M). Meropenem tablets (Oxoid Ltd., London, England, # CT0774B) were incubated at 35°C for 4 h ± 15 min and then placed on MH agar (Hope Bio-Technology Co., Ltd., Qingdao, #HB6231) inoculated with carbapenem-sensitive *E. coli* ATCC 25922. After 16–20 h of incubation, the mCIM results were interpreted as follows: an inhibition zone of ≥19 mm = negative; 6–15 mm = positive; and 16–18 mm with a tip-sized colony = intermediate (positive). A ≥5 mm increase in the eCIM inhibition zone compared to the mCIM indicates a positive result for metallo-carbapenemase; an increase of <4 mm indicates a negative result for metallo-carbapenemase. *Escherichia coli* ATCC 25922 served as the control. All experiments were conducted in triplicate.

### DNA extraction and detection of antibiotic sensitivity tests

DNA extraction was performed using the heating extraction method as previously described (Zhang et al., 2021b), and the extracted DNA was stored at −80°C. All isolates were assessed for antibiotic sensitivity to 11 antibiotics, following the methodology recommended by CLSI. In brief, bacterial cultures were prepared and dispensed into sterile 96-well polystyrene plates at a volume of 200 μL per well. The concentrations of antibiotics in wells 1 to 11 were 512, 256, 128, 64, 32, 16, 8, 4, 2, 1, and 0.5 mg/L, respectively. The 12th well served as a blank control containing Mueller-Hinton medium without antibiotics. The 11 antibiotics tested included imipenem (IMP, #S24020), tetracycline (TE, #S17051), norfloxacin (NFX, #S17080) (Yuanye Biotechnology Co., Ltd., Shanghai), azithromycin (AZM, #MB1024), kanamycin (KAN, #MB1130), trimethoprim (SXT, #MB1317), polymyxin B (PB, #MB1188) (Meilun Biotechnology Co., Ltd., Dalian), chloramphenicol (C, #IC0320) (Solarbio Science & Technology Co., Ltd., Beijing), aztreonam (ATM, #A801653), cefotaxime (CTX, #C804340), and ampicillin (AMP, #A830931) (Macklin Biochemical Technology Co., Ltd., Shanghai). *Escherichia coli* ATCC25922 was utilized as the negative control strain.

### Screening for ARGs in *Escherichia coli* isolates

Based on the MIC results, it was necessary to detect the presence of ARGs in the isolates. PCR was used to detect ARGs associated with different classes of antibiotics, including *β*-lactams (*bla*_TEM_, *bla*_SHV_, *bla*_CTX-M_, *bla*_NDM_, *bla*_KPC_), quinolones (*qnrA*, *qnrB*, and *qnrS*), aminoglycosides [*aac(6′)-Ib-cr*, *aadA1*, and *aac(3)-I*], sulfonamides (*sul1*, *sul2*, and *sul3*), tetracyclines (*tet*A, *tet*B, *tet*C, and *tet*M), glucocorticoids (*floR*, *cat1*), macrolides (*ermA*, *ermB*), and polymyxin (*mcr-1*). The primers used for PCR are detailed in Supplementary Table S1. All positive PCR products were sent to Tsingke Biotechnology Co., Ltd. for DNA sequencing. The resulting sequences were analyzed using the BLAST tool available at the online gene database.^1^

### Conjugational transfer and conjugational frequency

We used a *bla*_NDM_-5-positive *E. coli* strain as the donor and NaN_3_-resistant *E. coli* J53 as the recipient. The donor and recipient bacteria were cultured on LB agar plates containing 4 mg/L IMP, 150 mg/L NaN_3_, and 4 mg/L IMP + 150 mg/L NaN_3_, respectively. After incubating for 18 h in a constant temperature incubator at 37°C, we observed the results. Both the donor and recipient bacteria were combined in a new tube at a ratio of 1:4 and incubated at 37°C for 24 h, followed by a 10-fold dilution in sterile saline to achieve the appropriate concentration. A volume of 100 μL of the mixed bacterial solution was then plated on LB agar containing 4 mg/L IMP + 150 mg/L NaN_3_ and incubated at 37°C for 24 h. Single colonies from the 4 mg/L IMP + 150 mg/L NaN3 LB agar were selected for *bla*_NDM-5_ detection, and *bla*_NDM-5_-positive strains were identified as transconjugants.

For testing conjugational frequency, both donor and recipient bacteria were activated and diluted to an OD600 of 0.5. They were then mixed in a new tube at a ratio of 1:1 and incubated at 37°C for 24 h, followed by a 10-fold dilution with sterile saline to reach the desired concentration. Subsequently, 100 μL of the mixed bacterial solution was plated on LB agar containing 4 mg/L IMP + 150 mg/L NaN_3_, while 100 μL of *E. coli* J53 was spread on LB agar with 150 mg/L NaN_3_. Both were incubated at 37°C for 24 h. Single colonies on the 4 mg/L IMP +150 mg/L NaN_3_ agar were counted as transconjugants (designated as T), while single colonies on the 150 mg/L NaN_3_ agar were counted as receptors (designated as R). The conjugation frequency was calculated as T/R. All experiments were conducted in triplicate.

### Plasmid replicon typing

The PCR-based replicon typing (PBRT) technique was used to detect plasmid incompatibility groups in transconjugants, as previously described with specific primers (Carattoli et al., 2005; Johnson et al., 2007).

### S1-PFGE

*Salmonella* H9812 served as the reference strain for molecular quality standards. For the analyses, the *bla*_NDM-5_-positive strains were embedded in 1% SeaKem Gold Agarose (Lonza Group Co., Ltd.) and subjected to enzymatic digestion using S1 nuclease (Takara Biomedical Technology Co., Ltd., Beijing) at 37°C for 4 h. Electrophoresis was subsequently performed using 1% agarose gel under specific conditions: an operating voltage of 6 V/cm and a pulse time ranging from 6.76 s to 35.38 s, utilizing the CHEF DR III system for a total duration of 18 h at 14°C.

### Impact of imipenem on the stability of a plasmid harboring *bla*_NDM-5_

The transconjugants were cultured in LB broth with 4 mg/L of imipenem (IMP) added continuously, with transfers to fresh medium every 12 h. Stability was measured every 24 h. Bacteria were diluted 10-fold to the appropriate concentration using sterile saline, and 100 μL of the diluted bacteria was applied to LB agar containing 4 mg/L of IMP, as well as to regular LB agar. The plates were then incubated in a constant temperature incubator at 37°C for 18 h. The number of single colonies on the LB agar with 4 mg/L of IMP indicated the number of *bla*_NDM-5_-positive strains, while the number of single colonies on regular LB agar represented the total number of strains. The genetic stability of the *bla*_NDM-5_-positive plasmid was calculated as the ratio of *bla*_NDM-5_-positive strains to the total number of strains. All studies were performed in triplicate.

### Construction of the pET32a (+)-*bla*_NDM-5_ plasmid

To amplify the complete *bla*_NDM-5_ sequence, primers *bla*_NDM-5_-F (5′-CGCCATATGGCGATGGAATTGCCCAAT-3′) and *bla*_NDM-5_-R (5′-CGCGGATCCGCGTCAATGATGATGATGAGATGGCGCAGCTTGTC-3′) were employed, incorporating *Nde* I and *BamH* I restriction sites at their respective termini. The amplification reaction mixture had a total volume of 20 μL and included 10 μL of 2 × Taq PCR Green Mix, 1 μL of each primer (*bla*_NDM-5_-F and *bla*_NDM-5_-R), 2 μL of DNA, and 6 μL of ddH_2_O. The PCR cycling conditions were as follows: initial denaturation at 95°C for 15 s, annealing at 55°C for 15 s, and extension at 65°C for 1 min, for a total of 32 cycles. The PCR products were analyzed using 1% agarose gel electrophoresis (120 V for 20 min) and purified using a PCR purification kit (Tiangen Biotech Co., Ltd., Beijing). A digestion reaction was performed with 5 μL of the purified PCR product (*bla*_NDM-5_) and the pET32a(+) vector containing 6 × His. The ligation was allowed to proceed overnight at 4°C. After sequencing the recombinant plasmid and confirming its accuracy through BLAST alignment, the plasmid was transformed into *E. coli-*BL21(DE3) and *E. coli* DH5α.

### Expression of the NDM-5 protein and activity detection

*Escherichia coli*-BL21(DE3)-pET32a(+)-*bla*_NDM-5_ was cultured with an optical density (OD600) of 0.6, and 0.5 M isopropyl-*β*-D-thiogalactoside (IPTG) (Solarbio Science & Technology Co., Ltd., Beijing) was added for induction at 16°C overnight. The bacteria were then collected and centrifuged. The resulting pellet was resuspended in 0.05 M Tris–HCl solution and disrupted using ice bath ultrasound. Subsequently, purification was performed according to the Ni-NTA Beads 6FF kit (Changzhou Smart-Lifesciences Biotechnology Co., Ltd.). The purified samples were then analyzed using sodium dodecyl sulfate-polyacrylamide gel electrophoresis (SDS-PAGE) (Li L. et al., 2024).

To evaluate the changes in MIC values of the tested strains against imipenem in the presence of NDM-5 protein, a reaction system was prepared consisting of 30 μg/mL of total protein and 11 concentrations of imipenem (512 mg/L, 256 mg/L, 128 mg/L, 64 mg/L, 32 mg/L, 16 mg/L, 8 mg/L, 4 mg/L, 2 mg/L, 1 mg/L, and 0.5 mg/L). The reaction was incubated at 37°C in triplicate alongside a negative control group that lacked protein. The changes in OD600 of the strains were measured using a microplate reader every 4 h over a total period of 24 h (at 4 h, 8 h, 12 h, 16 h, 20 h, and 24 h) to determine alterations in MIC values against imipenem.

### Fitness cost of *bla*_NDM-5_-positive plasmids

To investigate the fitness associated with *bla*_NDM-5_-positive plasmids, we employed a combination of growth curve analyses, biofilm formation assessments, and *in vitro* competition assays. These experiments utilized the conjugants *E. coli* DH5α-pET32a(+)-*bla*_NDM-5_ and *E. coli* DH5α-pET32a(+), following previously described methods (Lu et al., 2021; Huo et al., 2024). All experiments were conducted in triplicate.

### Exposure to varying doses of imipenem

A low-level imipenem-resistant strain carrying *bla*_NDM-5_ was selected and cultured to an OD600 of 1. Based on the MIC results for imipenem, a concentration of 1 × MIC was chosen for induction on the first day. The concentration of imipenem was doubled daily, following the sequence: 1 × MIC, 2 × MIC, 4 × MIC, 8 × MIC, and so on. Each day, 500 μL of the culture was transferred to 20 mL of LB broth supplemented with the corresponding concentration of imipenem, and the culture was incubated at 37°C for 24 h until no further growth was observed.

### Real-time PCR (qRT-PCR)

The expression of *bla*_NDM-5_ in high-level resistant strains after induction was assessed using qRT-PCR. The fluorescent quantitative primers for *bla*_NDM-5_ were selected based on a previous study (Zhao et al., 2023). The TAKARA RNAiso Easy Kit (Takara Biomedical Technology Co., Ltd., Beijing) was used to isolate mRNA from imipenem-exposed strains, and the Hifair ® II 1st Strand cDNA Synthesis SuperMix Kit (Yeasen Biotechnology Co., Ltd., Shanghai) was used for reverse transcription to synthesize cDNA. The expression levels of *bla*_NDM-5_ were quantified using cDNA as a template, with the 16S rRNA gene serving as the internal reference. The results were expressed as the ratio of the expression levels of the *bla*_NDM-5_ target gene compared to the internal reference gene (Zhao et al., 2023).

### MIC determination of antibiotic-resistant strains post-induction with the addition of efflux pump inhibitors

The MIC of imipenem was determined using the broth microdilution method, with 5 mg/L of carbonyl cyanide m-chlorophenylhydrazine (CCCP) (Solarbio Science & Technology Co., Ltd., Beijing) as an efflux pump inhibitor. This method followed the same protocol as the antibiotic sensitivity test.

### Fitness cost and stability of induced strains

To study the adaptive cost and stability of the altered strains, a combination of growth curve analysis, *in vitro* competition tests, and stability assessments was employed. The experiment utilized strains both before and after induction, based on previous references (Lu et al., 2021; Huo et al., 2024). All experiments were conducted in triplicate.

### Statistical analysis

Statistical analyses were performed using SPSS 27.0 software. Pearson’s chi-square test and Fisher’s exact test were utilized to compare the antimicrobial resistance of 103 *E. coli* isolates and evaluate the correlation among different ARGs, or between AMR phenotypes and their corresponding ARGs. Odds ratios (OR) and their 95% confidence intervals were calculated, with OR < 1 indicating a negative correlation and OR > 1 indicating a positive correlation. A *p* value of <0.05 was considered statistically significant.

## Results

### Isolation and identification of *bla*_NDM-5_-positive *Escherichia coli* from waterfowl sources

Upon transporting ducks from selected areas to the laboratory, researchers collected intestinal contents in a clean and disinfected animal facility to ensure non-repetitive samples of intestinal feces from each duck. A total of 431 intestinal fecal samples were collected from each duck. From a total of 431 intestinal feces collected from waterfowl sources in Sichuan, Anhui, Shaanxi, Xinjiang, and Chongqing, 103 strains were identified as *bla*_NDM-5_-positive *E. coli*, resulting in a positive rate of 30.5%. The majority of *bla*_NDM-5_-positive *E. coli* were isolated from Sichuan Province, specifically from areas such as Deyang, Meishan, Xinjin, Chongzhou, Jintang, Suining, Dayi, and Leshan. Overall, with the exception of Shaanxi Province, the farms from which the positive bacterial strain samples were collected are located between 20 and 300 km from our laboratory. Specific details are provided in Table 1.

**Table 1:** Isolation of 103 *bla*_NDM-5_-positive *Escherichia coli.*

Region	Abbreviation of the region	Number of samples	*bla*_NDM-5_-positive *E. coli*	Isolation rates
Deyang	DY	92	28	30.4%
Meishan	MS	18	12	66.7%
Xinjin	XJ	22	18	81.8%
Chongzhou	CZ	19	15	79.0%
Jintang	JT	2	2	100.0%
Suining	SN	14	8	57.1%
Shaanxi	SX	14	13	92.9%
Dayi	dy	16	3	18.8%
Leshan	LS	5	4	80.0%

### Results of carbapenemase phenotype screening

All *bla*_NDM-5_-positive *E. coli* strains tested positive in the MHT, mCIM, and eCIM tests. The results of the MHT are presented in Supplementary Figure S1, while the mCIM and eCIM results are shown in Supplementary Table S2 and Supplementary Figure S2. Specific values can be found in the Appendix.

### Analysis of ARGs in *bla*_NDM-5_-positive *Escherichia coli* strains

**Figure 1:**
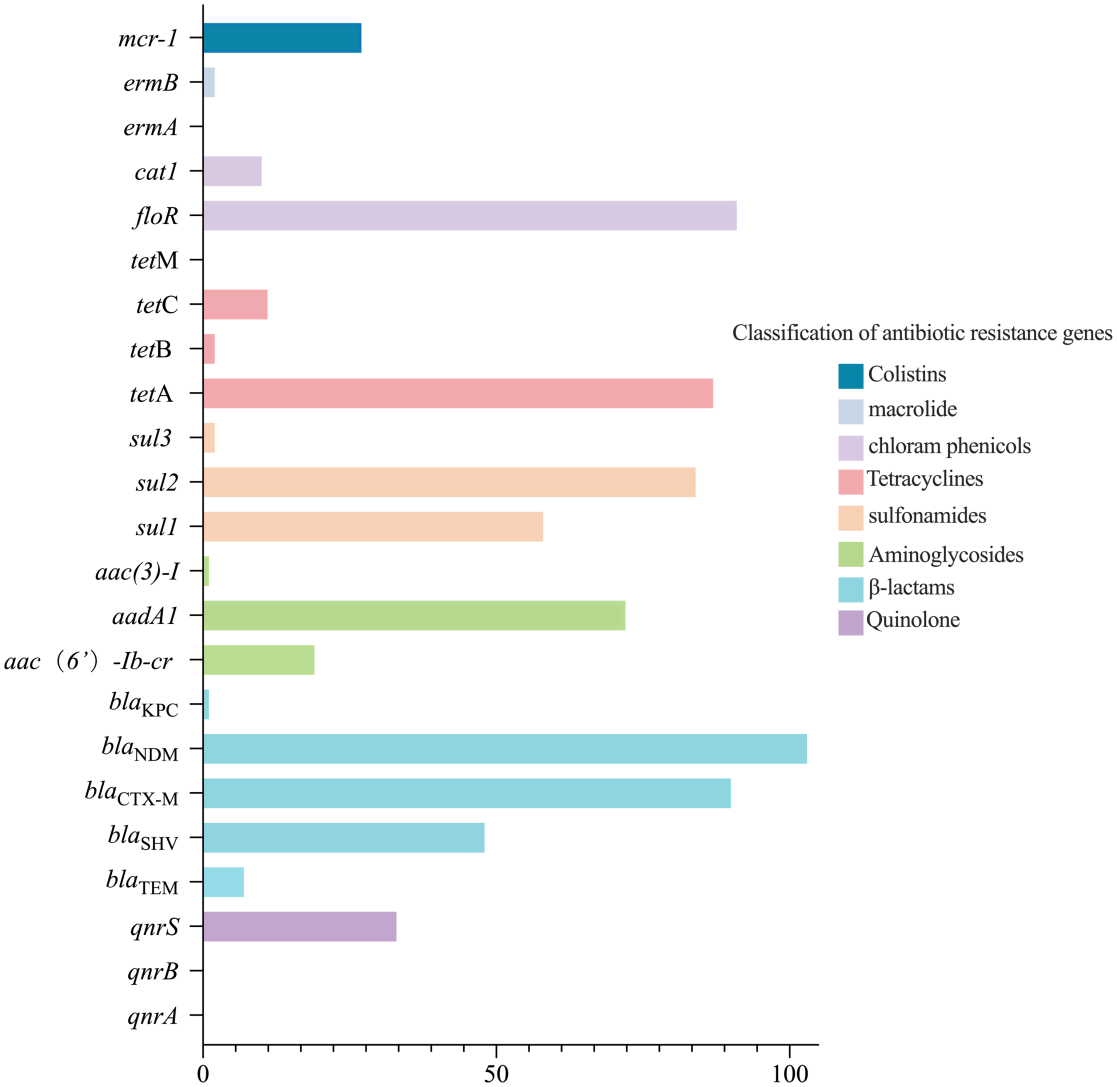
The result of ARGs detection in 103 *bla*_NDM-5_-positive *E. coli*.

The detection of ARGs in 103 *bla*_NDM-5_-positive *E. coli* strains is presented in Figure 1. Among these, the chloramphenicol resistance gene *floR* had the highest detection rate (*n* = 91, 88.3%), followed by the *β*-lactam resistance gene *bla*_CTX-M_ (*n* = 90, 87.4%), the tetracycline resistance gene *tet*A (*n* = 87, 84.5%), the sulfonamide resistance gene *sul2* (*n* = 84, 81.6%), the aminoglycoside resistance gene *aadA1* (*n* = 72, 69.9%), and the sulfonamide resistance gene *sul1* (*n* = 58, 56.3%). The detection rates of the other ARGs were less than 50%.

The analysis results indicate that there are significant correlations, primarily positive, among the ARGs carried by the 103 *bla*_NDM-5_-positive *E. coli* strains. As shown in Supplementary Table S3, a total of 45 pairs of ARGs exhibited significant positive correlations, while 23 pairs showed significant negative correlations. Among these, *tet*B has the strongest positive correlation with *sul3* or *ermB* (OR, 101.000; 95% CI, 3.370–3027.447). In terms of negative correlations, *sul2* and *tetC* displayed the strongest negative correlation (OR, 0.030; 95% CI, 0.006–0.156). Detailed analyses of the other correlation results are presented in Supplementary Table S3.

### Antimicrobial susceptibility analysis

Except for strain DY51, all strains exhibited high-level resistance to imipenem, with MIC values ranging from 64 to ≥512 mg/L (Supplementary Table S4). The resistance rates for the remaining 10 antibiotics were as follows: AMP (*n* = 103, 100%), CTX (*n* = 102, 99.0%), and NFX (*n* = 100, 97.1%) showed the highest resistance rates, followed by SXT (*n* = 92, 89.3%), KAN (*n* = 92, 89.3%), and C (*n* = 92, 89.3%). Finally, the resistance rates for AZM (*n* = 28, 27.2%) and PB (*n* = 16, 15.5%) were relatively low.

These results indicate that all 103 *bla*_NDM-5_-positive *E. coli* strains are MDR, with resistance levels ranging from 3 to 8 antibiotics, primarily six antibiotics (*n* = 57, 55.3%). The specific antimicrobial resistance profiles are presented in Table 2.

**Table 2:** Multidrug resistance profile of 103 *bla*_NDM-5_-positive *Escherichia coli* strains.

Drug resistance spectrum	Number of strains
KAN + AZT + C + TET + CTX + TMP + NFX + AMP + IMP	44
KAN + AZT + C + TET + CTX + NFX + AMP + IMP	10
KAN + AZT + C + TET + CTX + TMP + AZM + NFX + AMP + IMP	9
KAN + C + TET + CTX + TMP + NFX + AMP + IMP	8
KAN + AZT + C + TET + PB + CTX + TMP + NFX + AMP + IMP	6
KAN + AZT + C + TET + PB + CTX + TMP + AZM + NFX + AMP + IMP	5
CTX + TMP + AZM + NFX + AMP + IMP	3
AZT + C + TET + CTX + TMP + AZM + NFX + AMP + IMP	2
KAN + AZT + C + TET + CTX + TMP + AMP + IMP	2
KAN + TET + CTX + TMP + NFX + AMP + IMP	2
AZT + C + TET + CTX + TMP + NFX + AMP + IMP	1
AZT + CTX + TMP + AZM + NFX + AMP + IMP	1
AZT + TET + PB + CTX + TMP + AZM + NFX + AMP + IMP	1
C + CTX + TMP + AZM + NFX + AMP + IMP	1
C + TET + PB + CTX + TMP + AZM + NFX + AMP + IMP	1
KAN + AZT + C + TET + CTX + TMP + AZM + AMP + IMP	1
KAN + AZT + C + TET + TMP + AZM + NFX + AMP + IMP	1
KAN + AZT + TET + CTX + TMP + AZM + NFX + AMP + IMP	1
KAN + AZT + TET + PB + CTX + TMP + AZM + NFX + AMP + IMP	1
KAN + C + TET + CTX + NFX + AMP + IMP	1
KAN + TET + PB + CTX + TMP + AZM + NFX + AMP + IMP	1
PB + CTX + TMP + NFX + AMP + IMP	1

The correlation analysis between resistance genes and AMR phenotypes revealed significant correlations between the resistance genes and AMR in the 103 *bla*_NDM-5_-positive *E. coli* strains from waterfowl, primarily negative correlations. As shown in Supplementary Table S5, a total of seven pairs of genes exhibited significant positive correlations, while nine pairs showed significant negative correlations. Among these, KAN and *aadA1* exhibited the strongest positive correlation (OR, 13.696; 95% CI, 2.757–68.036), while NFX and *emrB* displayed the strongest negative correlation (OR, 0.020; 95% CI, 0.001–0.446). Other analyses are detailed in Supplementary Table S5.

### Analysis of plasmid-mediated horizontal transfer of *bla*_NDM-5_

**Figure 2:**
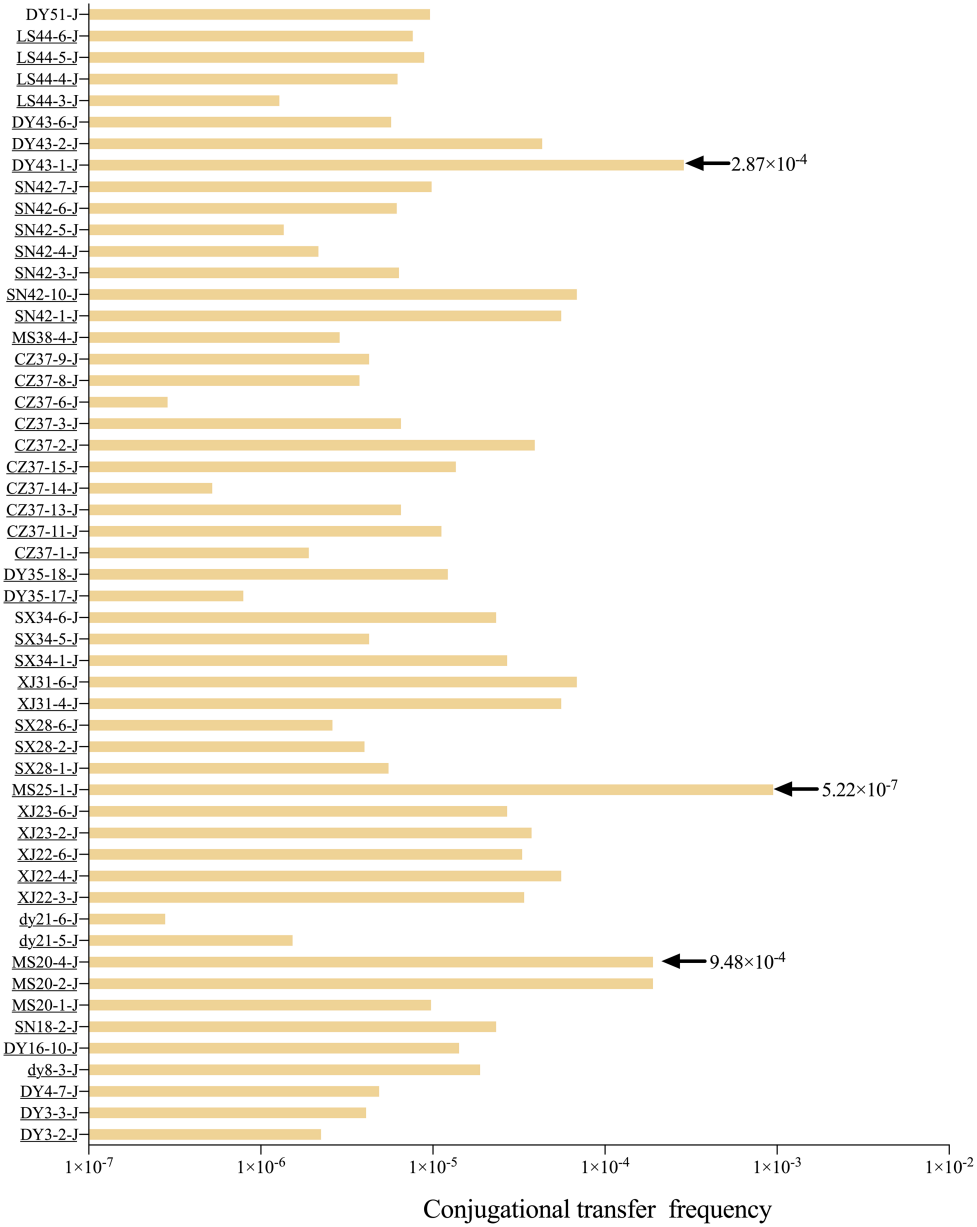
Conjugation frequency of 53 strains *bla*_NDM-5_-positive *E. coli* transconjugants.

Fifty-three *bla*_NDM-5_-positive *E. coli* strains were able to transfer imipenem resistance to the recipient strain J53, with the MIC of the recipient strain increasing from 0.5 mg/L to either 4 mg/L or 512 mg/L. PCR and DNA sequencing confirmed that all 53 transconjugants were *bla*_NDM-5_-positive. By counting the number of colonies of the 53 *bla*_NDM-5_-positive transconjugants on double-resistant plates and the colonies of *E. coli* J53 on NaN3-resistant plates, the range of conjugative transfer frequency was calculated to be 5.22 × 10^−7^ to 9.48 × 10^−4^ (Figure 2). The plasmid profiles of randomly selected donor and recipient bacteria were analyzed using S1-PFGE (Supplementary Figure S3). The donor bacteria carried 3–4 plasmids, while the recipient bacteria carried 1 plasmid, indicating that *bla*_NDM-5_ was located on a mobile plasmid. Furthermore, replicon typing of all transconjugants was performed via PCR and sequencing; the results indicated that the 53 *bla*_NDM-5_-positive transconjugants harbored 13 different replicon types, namely, IncX1, IncX3, HI2, I1, N, FIA, FIB, Y, P, FIC, FIIS, K, and B/O. Notably, IncX1 (28.3%) was the dominant type, followed by HI2 (26.4%) and K (15.1%). The plasmid profiles of randomly selected donor and recipient bacteria were shown by S1-PFGE (Supplementary Figure S3). The donor bacteria carried 3–4 plasmids, while most recipient bacteria carried one plasmid, indicating that *bla*_NDM-5_ was located on a mobile plasmid.

### The impact of imipenem on the stability of *bla*_NDM-5_-positive plasmids

**Figure 3:**
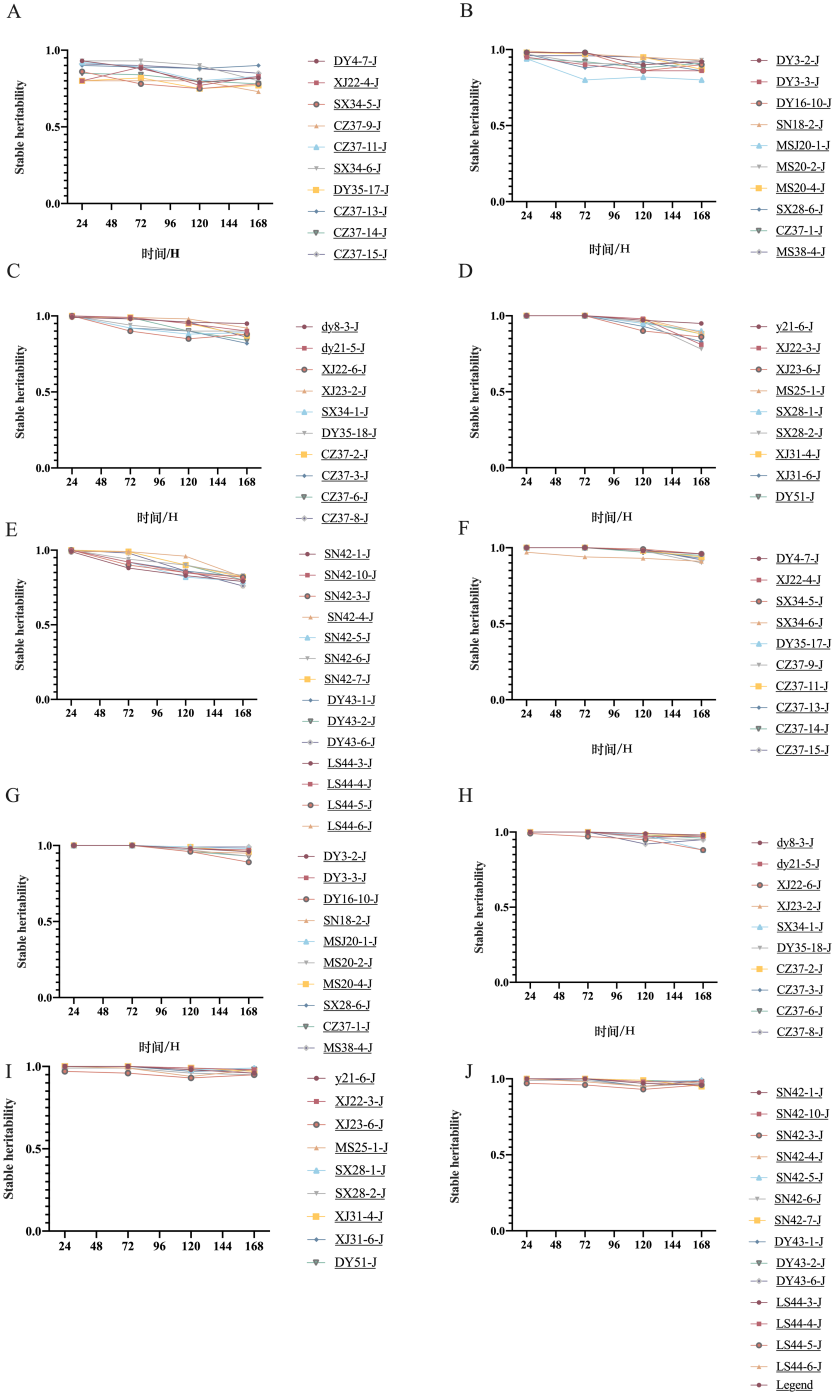
Plasmid genetic stability of 53 transconjugants with or without imipenem selection pressure.

To investigate whether the *bla*_NDM-5_-positive plasmids acquired by the transconjugants possess stable genetic characteristics, this study conducted a plasmid stability test on 53 strains carrying *bla*_NDM-5_-conjugative plasmids. During the experiment, the genetic stability of these plasmids was compared under conditions of both imipenem selection pressure and in its absence, as illustrated in Figure 3. In the absence of antibiotic selection pressure, the plasmid stability of the 53 transconjugants showed a slight decline with increasing passage numbers, while the plasmid stability rate remained above 75%. Under a selection pressure of 4 mg/L imipenem, the plasmid stability rate of the 53 transconjugants was even higher than that observed without antibiotics, maintaining above 92%, indicating stronger stability. This suggests that the stable presence of *bla*_NDM-5_ conjugative positive plasmids is influenced not only by the stable genetic characteristics of the gene itself but also by the presence of antibiotics in the environment. These findings imply that residual antibiotics in the environment can contribute to the stable presence of *bla*_NDM-5_-positive plasmids in resistant strains, thereby enhancing the dissemination of antibiotic resistance.

### Construction results of the pET32a (+)-*bla*_NDM-5_ recombinant plasmid

Full-length amplification of *bla*_NDM-5_ was performed on the transformant *E. coli* DH5α carrying the pET32a(+)-*bla*_NDM-5_ plasmid, and the electrophoresis gel results are presented in Supplementary Figure S4. The band size is approximately 813 bp, which is consistent with the expected size. The amplification product was sequenced, and the sequencing results were subjected to BLAST alignment, confirming the successful construction of the recombinant plasmid.

### Expression and activity detection of NDM-5 protein

Total protein was extracted from the modified *E. coli*-BL21(DE3) strain harboring the pET32a(+)-*bla*_NDM-5_ plasmid. The *bla*_NDM-5_ protein was subsequently purified using nickel column affinity chromatography. After SDS-PAGE analysis, a clear protein band was observed at the 28.5 kDa position, as shown in Supplementary Figure S5, aligning with the expected size of the NDM-5 protein.

**Figure 4:**
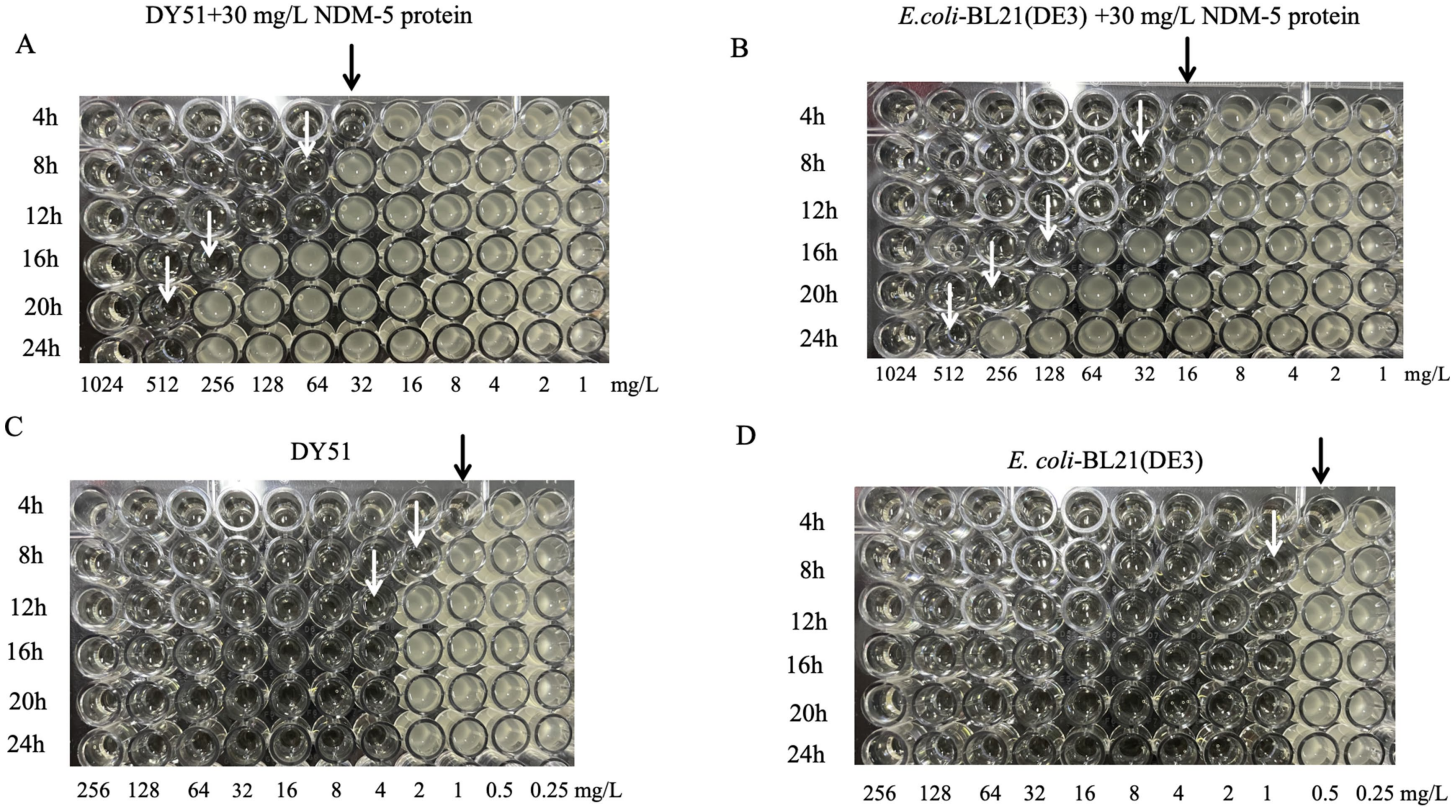
The change of MIC value of imipenem to DY51 and *E. coli*-BL21(DE3) with or without purified NDM-5 protein. Note: Each well in Figures **A** and **B** contains 30mg/L purified NDM-5 protein, while each well in Figures C and D does not contain purified NDM-5 protein.

Initially, the concentration of the purified NDM-5 protein was determined. Based on this concentration, the activity of the purified NDM-5 protein was assayed. Table 3 and Figure 4 described the changes in the MIC values of imipenem against the low-level resistant strain DY51 in the presence of NDM-5 protein. Due to the hydrolytic activity of the NDM-5 protein on imipenem, the resistance of the low-level resistant strain DY51 and the non-*bla*_NDM-5_-carrying strain *E. coli*-BL21(DE3) to imipenem gradually increased, resulting in rising MIC values over time. This clearly demonstrates that the purified NDM-5 protein exhibits significant activity, capable of hydrolyzing imipenem and enabling the strains to survive in the presence of high antibiotic concentrations.

**Table 3:** Change of MIC value of imipenem to DY51 in the presence of NDM-5 protein.

Test strain	NDM-5 protein (30 μg/mL)	MIC(μg/mL)					
		4 h	8 h	12 h	16 h	20 h	24 h
DY51	*+*	32	64	64	256	512	512
*E. coli*-BL21(DE3)	*+*	16	32	32	128	256	512
DY51	−	1	2	4	4	4	4
*E. coli*-BL21(DE3)	−	0.5	1	1	1	1	1

### The impact of *bla*_NDM-5_-positive plasmids on recipient bacteria

**Figure 5:**
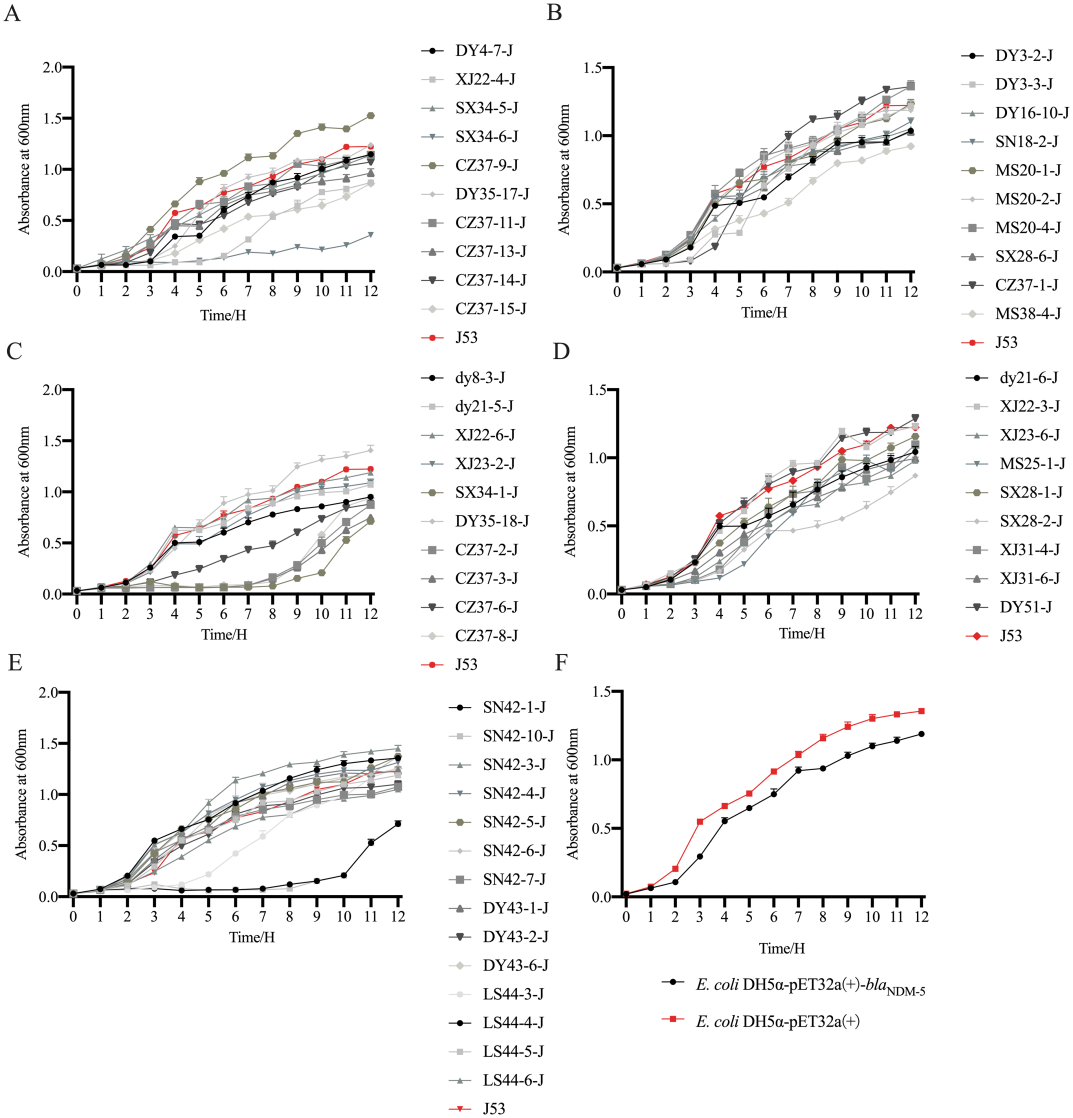
Growth curve of *bla*_NDM-5_ transconjugants and transformant strains. Note: Figure **A**, **B**, **C**, **D**, **E** are the growth curve of *bla*_NDM-5_ transconjugants, and Figure **F** is the growth curve of *bla*_NDM-5_ transformants.

The OD_600_ values of 53 transconjugants, the transformant *E. coli* DH5α-pET32a(+)-*bla*_NDM-5_, *E. coli* DH5α-pET32a(+), and the recipient bacteria *E. coli* J53 were measured at various time points to plot the changes in growth curves before and after the transfer of *bla*_NDM-5_-positive plasmids. The results are presented in Figure 5. Among the 53 transconjugants, 32 of them (DY3-2-J, DY4-7-J, dy8-3-J, SN18-2-J, dy21-5-J, dy21-6-J, XJ22-4-J, XJ22-6-J, XJ23-2-J, XJ23-6-J, MS25-1-J, SX28-1-J, SX28-2-J, SX28-6-J, XJ31-4-J, XJ31-6-J, SX34-1-J, CZ37-2-J, CZ37-3-J, CZ37-6-J, CZ37-8-J, CZ37-11-J, CZ37-13-J, CZ37-14-J, CZ37-15-J, SX34-6-J, MS38-4-J, SN42-1-J, SN42-7-J, DY43-2-J, LS44-4-J, and LS44-5-J) exhibited slower growth rates after acquiring the *bla*_NDM-5_-positive plasmid, while the growth rates of the remaining 21 transconjugants (DY3-3-J, MS20-1-J, MS20-2-J, MS20-4-J, XJ22-3-J, SX34-5-J, DY35-17-J, DY35-18-J, CZ37-1-J, CZ37-9-J, SN42-10-J, SN42-3-J, SN42-4-J, SN42-5-J, SN42-6-J, DY43-1-J, DY43-6-J, LS44-3-J, LS44-6-J, DY16-10-J, and DY51-J) were comparable to that of *E. coli* J53. In the transformant group, the growth rate of *E. coli* DH5α-pET32a(+)-*bla*_NDM-5_, which acquired the *bla*_NDM-5_ gene, was slower compared to *E. coli* DH5α-pET32a(+).

**Figure 6:**
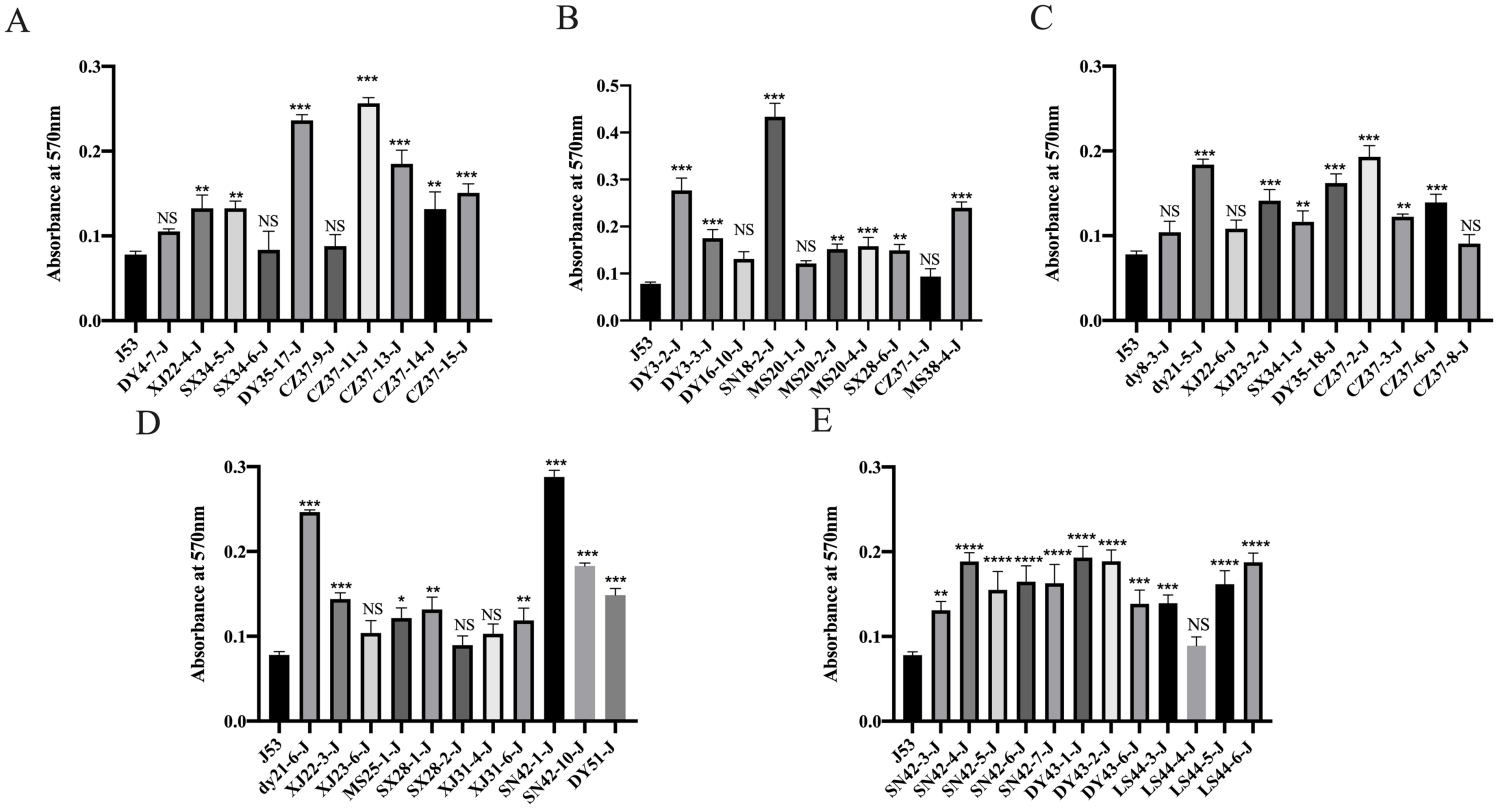
Results of biofilm formation ability of 53 strains of *bla*_NDM-5_ transconjugants Note: Figure **A**, **B**, **C**, **D** and **E** are the results of biofilm formation ability of different *bla*_NDM-5_ transconjugants.

The biofilm formation ability of the 53 *bla*_NDM-5_ transconjugants and *E. coli* J53 was assessed by measuring the OD value at a wavelength of 570 nm. A higher OD_570_ value indicates stronger biofilm formation ability. The results are displayed in Figure 6. Among the 53 *bla*_NDM-5_ transconjugants, 40 transconjugants (XJ22-4-J, SX34-5-J, DY35-17-J, CZ37-11-J, CZ37-13-J, CZ37-14-J, CZ37-15-J, DY3-2-J, DY3-3-J, SN18-2-J, MS20-2-J, MS20-4-J, SX28-6-J, MS38-4-J, dy21-5-J, XJ23-2-J, SX34-1-J, DY35-18-J, CZ37-2-J, CZ37-3-J, CZ37-6-J, dy21-6-J, XJ22-3-J, MS25-1-J, SX28-1-J, XJ31-6-J, SN42-1-J, SN42-10-J, DY51-J, SN42-3-J, SN42-4-J, SN42-5-J, SN42-6-J, SN42-7-J, DY43-1-J, DY43-2-J, DY43-6-J, LS44-3-J, LS44-5-J, and LS44-6-J) demonstrated significantly enhanced biofilm formation ability after acquiring the *bla*_NDM-5_ conjugative plasmid. In contrast, 13 transconjugants (DY4-7-J, SX34-6-J, CZ37-9-J, DY16-10-J, MS20-1-J, CZ37-1-J, DY8-3-J, XJ22-6-J, CZ37-8-J, XJ23-6-J, SX28-2-J, XJ31-4-J, and LS44-4-J) showed no significant difference in biofilm formation ability compared to the recipient bacteria *E. coli* J53 after acquiring the *bla*_NDM-5_ plasmid.

To investigate the effect of *bla*_NDM-5_ on the fitness of transformants, an *in vitro* competition experiment was conducted between *E. coli* DH5α-pET32a(+)-*bla*_NDM-5_ and *E. coli* DH5α-pET32a(+). As shown in Supplementary Figure S6, the relative fitness (RF) values were less than 1 at 24, 48, and 72 h, indicating that the acquisition of *bla*_NDM-5_ imposed a certain fitness cost on the transformants, although this cost was relatively small.

### *In vitro* induction of resistance to imipenem in *Escherichia coli*

Using MIC testing, this study identified a strain DY51 with low-level resistance to imipenem (MIC = 4 mg/L). Furthermore, the recombinant strain *E. coli* DH5α-pET32a(+)-*bla*_NDM-5_ constructed in this study also exhibited a MIC value of 4 mg/L for imipenem. Based on these results, the low-level resistant strains DY51 and *E. coli* DH5α-pET32a(+)-*bla*_NDM-5_ were selected for an *in vitro* antibiotic induction experiment with imipenem. After exposure to varying doses of imipenem, two high-level imipenem-resistant strains, namely, DY51-I and *E. coli* DH5α-pET32a(+)-*bla*_NDM-5_-I, were obtained. The MIC value of strain DY51-I for imipenem increased to 512 mg/L, representing a 128-fold increase. Similarly, the MIC value of *E. coli* DH5α-pET32a(+)-*bla*_NDM-5_-I for imipenem also rose to 512 mg/L, also a 128-fold increase.

### Results of *bla*_NDM-5_ gene expression in the exposed resistant strains

**Figure 7:**
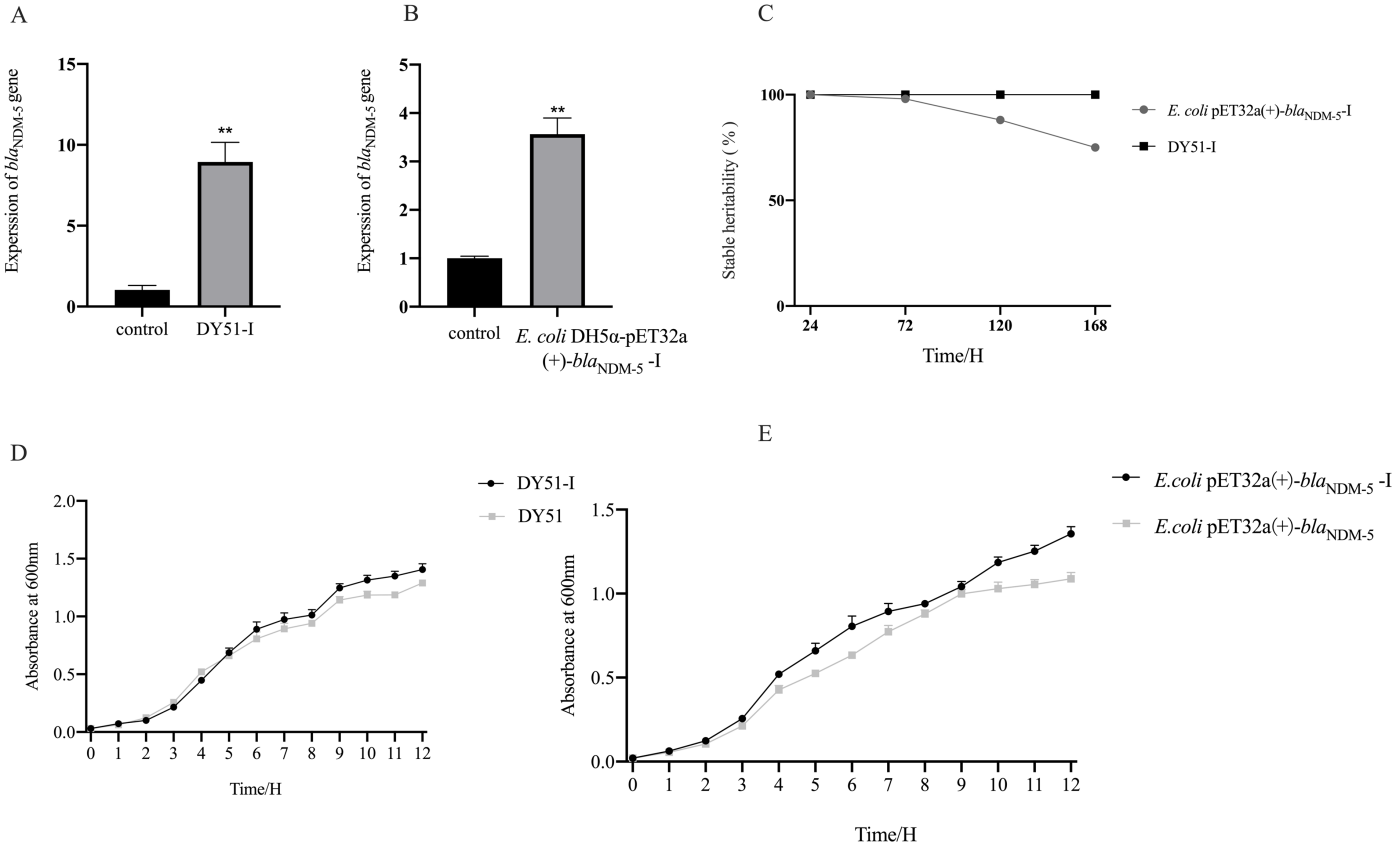
It shows the gene expression, genetic stability, and growth curve results of imipenem exposed strains, demonstrating their ability to adapt to antibiotic stress environments. Note: Figure **A** and **B** are the relative expression of *bla*_NDM-5_ gene, Figure **C** shows the genetic stability of *bla*_NDM-5_-positive plasmid in induced strains, Figure **D** is the growth curve comparison before and after DY51 induction, and Figure **E** is the growth curve comparison before and after *E.coli* DH5ɑ-pET32a(+)-*bla*_NDM-5_ induction.

Since there is a significant relationship between the expression level of the *bla*_NDM-5_ gene and bacterial resistance, qRT-PCR was utilized to determine the expression levels of the *bla*_NDM-5_ gene in *E. coli* DH5α-pET32a(+)-*bla*_NDM-5_ and DY51 strains before and after induction. As illustrated in Figures 7A,B, the expression level of the *bla*_NDM-5_ gene in DY51 increased following exposure, with the expression level in the exposed resistant strain DY51-I rising approximately 8-fold compared to the pre-exposed strain, showing a significant difference (*p* < 0.05). Similarly, the *bla*_NDM-5_ expression level in the exposed resistant strain *E. coli* DH5α-pET32a(+)-*bla*_NDM-5_-I increased approximately 4-fold in comparison with the pre-changed strain, also showing a statistically significant difference (*p* < 0.05). This suggests that the high-level resistance to imipenem in the exposed resistant strains *E. coli* DH5α-pET32a(+)-*bla*_NDM-5_-I and DY51-I may be attributed to a substantial increase in the expression level of the *bla*_NDM-5_ gene.

### MIC of imipenem-exposed resistant strains with efflux pump inhibitors

Overexpression of efflux pumps can decrease the sensitivity of *E. coli* to imipenem. Therefore, the MICs of the highly imipenem-resistant changed strains DY51-I and *E. coli* DH5α-pET32a(+)-*bla*_NDM-5_-I were determined following the addition of the efflux pump inhibitor CCCP. As indicated in Table 4, the MIC values of the highly imipenem-resistant changed strains decreased in the presence of CCCP, suggesting that the high resistance to imipenem in these strains is related to the overexpression of efflux pumps.

**Table 4:** MIC value of drug resistance changed strains with or without CCCP was added.

Test strain	Imipenem (mg/L)	Imipenem+CCP (mg/L)	Reduce multiples
DY51-I	512	128	4
*E. coli*-BL21(DE3)-*bla*_NDM-5_-I	512	128	4

### Stability and adaptability of *in vitro*-induced bacterial strains

To investigate the stability of high-level imipenem resistance in induced strains, resistance stability tests were conducted on the induced resistant strains *E. coli* DH5α-pET32a(+)-*bla*_NDM-5_-I and DY51-I. As shown in Figure 7C and Supplementary Table S6, the MIC values of the induced strains *E. coli* DH5α-pET32a(+)-*bla*_NDM-5_-I and DY51-I against imipenem remained at 512 mg/L. However, it is noteworthy that under conditions without antibiotic pressure, the induced strain *E. coli* DH5α-pET32a(+)-*bla*_NDM-5_-I gradually lost the *bla*NDM-5-positive plasmid. In contrast, the high resistance to imipenem in the induced strain DY51-I could be stably maintained for a considerable period without antibiotic pressure.

To investigate the growth characteristics of the induced resistant strains, growth curve experiments were performed on *E. coli* DH5α-pET32a(+)-*bla*_NDM-5_-I and DY51-I. The results, as shown in Figures 7D,E, indicate that both induced resistant strains exhibited faster growth rates compared to their non-changed counterparts.

Pairwise *in vitro* competition experiments were conducted between the high-level resistant strains *E. coli* DH5α-pET32a(+)-*bla*_NDM-5_-I and DY51-I and their pre-induced counterparts *E. coli* DH5α-pET32a(+)-*bla*_NDM-5_ and DY51. As shown in Supplementary Figure S10, after 24 h, the high-resistant strains *E. coli* DH5α-pET32a(+)-*bla*_NDM-5_-I and DY51-I displayed relatively strong competitive advantages over the pre-induced strains. However, at 48 h, the high-resistant strains demonstrated absolute competitive superiority.

## Discussion

Although carbapenem antibiotics are approved only for human clinical treatment, resistance to them has been detected in bacteria from environmental, animal, and food sources, posing a challenge to clinical practice. The emergence and prevalence of bacteria carrying the *bla*_NDM_ gene significantly increase the risk of bacterial resistance to carbapenem antibiotics while also seriously affecting the efficacy of *β*-lactam antibiotics. This represents a major challenge in clinical anti-infective treatment. In this study, 431 intestinal fecal samples were collected from waterfowl in Sichuan, Anhui, Shaanxi, Xinjiang, and Chongqing. The positive isolation rate of *bla*_NDM-5_-positive *E. coli* was found to be 30.5%. Strains carrying the *bla*_NDM_ gene have been isolated from animal feces, food, and environmental sources across China, with *bla*_NDM-5_ being the predominant carrier type found in animals. *Bla*_NDM-5_-positive strains have also been isolated from the feces of pigs, chickens, seagulls, and bar-headed geese in China. Interestingly, *E. coli* carrying *bla*_NDM-5_ was also isolated from neonatal patients in Sichuan province (Bai et al., 2023; Wen et al., 2023; Wu et al., 2023).

Antibiotic-resistant genes have been detected in various environmental matrices, including surface water, hospital wastewater, groundwater, urban wastewater, soil, manure, and air. These matrices interact with humans and animals, accelerating the spread of ARGs. Therefore, ARGs are considered as a new type of environmental pollutant (Tan et al., 2018; Han et al., 2022). In this study, ARGs were detected in 103 *bla*_NDM-5_-positive *E. coli* strains, resulting in the identification of a total of 19 ARGs. Among them, *floR* had the highest detection rate at 88.3%, followed by *bla*_CTX-M_ (87.4%) and *tet*A (84.5%). Compared with the results of ARG detection in *E. coli* isolated from waterfowl in select regions of China between 2021 and 2022 (Zhang et al., 2023), the types of ARGs detected in this study were similar to previous studies; however, the detection rate of ARGs in *bla*_NDM-5_-positive *E. coli* was found to be higher. The overuse or improper use of antibiotics may exacerbate the spread of ARGs and the development of bacterial resistance. Based on the ARGs with the highest detection rates, it is speculated that tetracyclines, sulfonamides, and *β*-lactam antibiotics have been widely used in the waterfowl breeding industry in the selected regions of this study. Notably, the simultaneous detection of *mcr-1* and *bla*_NDM_ in a single bacterium poses a threat to the treatment of multidrug-resistant bacteria, thus representing significant risks to the healthy development of livestock and poultry breeding. This threat has been evident since the discovery of plasmid-mediated *mcr-1* in *E. coli* isolated from humans and pigs in China in 2015 (Liu et al., 2016), when the effectiveness of antibiotics was significantly reduced. Many studies have reported the coexistence of *mcr-1* and *bla*_NDM_ (Cao et al., 2021; Abe et al., 2022; Kuang et al., 2022; Wang et al., 2023). In addition, in this study, we conducted computational statistical analyses to examine correlations among ARGs as well as between AMR phenotypes and ARGs. The results revealed significant correlations among ARGs as well as between AMR phenotypes and ARGs. However, it is crucial to acknowledge that the correlation among AMR genes is somewhat compromised by the lack of whole-genome sequencing data, including precise plasmid sequence data, which should be a focus of future research.

Increasing research indicates that *bla*_NDM_-positive *E. coli* frequently exhibits resistance to several antibiotics. Consequently, the microbroth dilution method was employed to determine the MIC of 11 antibiotics among 103 strains. Except for DY51, all strains demonstrated high-level resistance to imipenem with MIC values ranging from 64 to ≥512 mg/L. The highest resistance rates were observed for AMP at 100%, CTX at 99.0%, and NFX at 97.1%. In contrast, the lowest resistance rates were noted for AZM at 27.2% and PB at 15.5%. Notably, despite the Chinese government’s ban on the use of chloramphenicol in all food-producing animals since 2002, the resistance rate to chloramphenicol in this study remains high, consistent with previous findings (Li et al., 2013; Mehanni et al., 2023; Qin et al., 2023). The continued use of other antibiotics within the chloramphenicol family, such as florfenicol and thiamphenicol, maybe a significant contributing factor. In addition, the prevalence of multidrug resistance among *E. coli* strains in this study is alarming, with all strains exhibiting multidrug resistance characterized by resistance to between three and eight different antibiotics, predominantly sextuple resistance (55.3%). Thus, continuous monitoring of bacterial resistance and stringent regulation of antibiotic usage are essential to curb the spread of antibiotic resistance and safeguard human health as well as ecological balance.

Horizontal gene transfer is a key pathway for bacteria to acquire exogenous ARGs and propagate these genes genetically, with plasmids playing a crucial role in facilitating this process (Carattoli, 2013; Hasegawa et al., 2018; Bethke et al., 2020). In this study, the transferability of *bla*_NDM-5_-positive *E. coli* was assessed through conjugation transfer experiments involving 103 *bla*_NDM-5_-positive strains. A total of 53 *bla*_NDM-5_-positive strains successfully underwent conjugation, resulting in a success rate of 51.5%. Compared to the recipient strain *E. coli* J53, the MIC values of the conjugants toward imipenem increased by 8–1,024 times, and the *bla*_NDM-5_ gene was detected in all 53 conjugants. The results from S1-PFGE indicated that donor strains harbored 3–4 plasmids, while most conjugants carried just one plasmid, strongly suggesting that the *bla*_NDM-5_ gene was transferred horizontally via plasmids. For donor strains that failed to successfully conjugate, it is speculated that the *bla*_NDM-5_ gene may be located on non-conjugative plasmids or integrated within the chromosome, leading to ineffective transfer. The conjugation frequency of plasmids reflects their ability to undergo horizontal transfer. In this study, the conjugation frequency of the 53 transconjugants ranged from 5.22 × 10^−7^ to 9.48 × 10^−4^, comparable to, or even slightly higher than, previously reported conjugation frequencies of plasmids carrying *bla*_NDM-5_ (Potron et al., 2011; Huang et al., 2015; Dong et al., 2022; Lv et al., 2022). Therefore, *bla*_NDM-5_-positive plasmids can efficiently spread in waterfowl-derived *E. coli*, potentially contributing further to the prevalence of *bla*_NDM-5_.

Plasmids typically confer potential benefits to bacteria, but they also impose certain burdens on the host, which can manifest as reduced growth rates and decreased competitiveness in strains carrying plasmids (Carattoli, 2013; San Millan et al., 2016). The adaptive costs associated with plasmids and the potential loss of plasmids during host cell division can indeed limit the widespread dissemination of plasmids within bacterial communities. However, it is perplexing that, regardless of whether the adaptive costs of plasmids to the host bacteria are high or low, they can persist in host bacteria for extended periods (Wein et al., 2019; Weiss et al., 2023). In this study, some *bla*_NDM-5_-positive plasmids were gradually lost during continuous subculturing without antibiotic pressure, while all strains carrying *bla*_NDM-5_-positive plasmids remained stable in the population under antibiotic-selective pressure. Plasmids can stably exist in bacterial populations due to intrinsic factors such as high-copy plasmids, medium and low-copy plasmids, and plasmid addiction systems (Harrison and Brockhurst, 2012; San Millan et al., 2016; Tsang, 2017). In addition, changes to plasmid-encoded traits, such as the regulation of plasmid replication and copy number, as well as the acquisition of beneficial genes, may play a role (San Millan et al., 2014; Stalder et al., 2017). Compensatory mutations and compensatory evolution can also help mitigate the adaptive costs imposed by plasmids (Harrison and Brockhurst, 2012; MacLean and San Millan, 2015; Porse et al., 2016). These factors help explain the stable existence of strains carrying *bla*_NDM-5_-positive plasmids under antibiotic pressure.

Although plasmids can provide potential benefits to host bacteria under certain conditions, such as exogenous DNA, they can also influence the regulation of bacterial energy networks or exhibit cytotoxic effects. These processes often impose adaptive costs on host bacteria, leading to decreased competitiveness when competing with other strains (San Millan and MacLean, 2017; Prensky et al., 2021). In this study, to assess the fitness cost associated with plasmids carrying *bla*_NDM-5_, the *bla*_NDM-5_ gene was cloned into the plasmid pET32a(+), resulting in the construction of the transformant *E. coli*-DH5α-pET32a(+)-*bla*_NDM-5_. Subsequently, this transformant was subjected to fitness cost evaluation alongside 53 conjugants and *E. coli* J53. In the growth curve experiment, the transformant *E. coli*-DH5α-pET32a(+)-*bla*_NDM-5_ and over half of the conjugants exhibited a reduction in growth rate after acquiring the *bla*_NDM-5_-positive plasmid. This may be related to the varying adaptability of different strains to the *bla*_NDM-5_-positive plasmid. In the *in vitro* competition experiment, the competitiveness of the transformant *E. coli*-DH5α-pET32a(+)-*bla*_NDM-5_ was slightly reduced, indicating that while the *bla*_NDM-5_-positive plasmid can weaken the competitiveness of the host bacteria, the fitness cost is relatively small. In the biofilm formation experiment, all conjugants exhibited an increase or no significant change in their biofilm formation ability after acquiring the *bla*_NDM-5_-positive plasmid. The formation of biofilms is influenced by different bacterial species and various external environmental factors. Biofilms can enhance bacterial resistance to harsh environments and increase their tolerance to antibiotics (Ramamurthy et al., 2022; Michaelis and Grohmann, 2023). The phenomenon of enhanced biofilm formation associated with the p3R-IncX3 plasmid carrying *bla*_NDM-5_ has been documented, indicating that it can boost the biofilm formation ability of certain host bacteria (Ma et al., 2020). Similarly, the IncHI2 plasmid carrying *oqxAB* has been reported to enhance biofilm formation in host bacteria (Shi et al., 2018). Although the co-integrating plasmid pSL131-IncA/C-IncX3 carrying *bla*_NDM-1_ reduces the growth rate of the host bacteria, it also increases their biofilm formation ability (Liu et al., 2021). Therefore, the enhanced biofilm formation ability in host bacteria due to *bla*_NDM-5_-positive plasmids may not only facilitate the dissemination of the *bla*_NDM-5_ gene but also complicate clinical treatment efforts. Based on this analysis, it appears that *bla*_NDM-5_-positive plasmids impose a certain fitness cost on the host bacteria; however, this cost is relatively minor. Consequently, in-depth research into the molecular transmission characteristics of *bla*_NDM-5_ and the effects of *bla*_NDM-5_-positive plasmids on host bacteria is crucial for monitoring the dissemination and prevalence of *bla*_NDM-5_. Such research will aid in developing effective prevention and control strategies to combat the spread of antibiotic resistance.

The frequent use of antibiotics plays a pivotal role in the development of bacterial resistance. Long-term reliance on antibiotics exerts continuous selective pressure on bacteria, prompting them to adapt and develop resistance through a series of complex mechanisms (Jernberg et al., 2010). In this study, *in vitro* antibiotic induction experiments revealed that DY51 could transition from low-level resistance (MIC = 4 mg/L) to high-level resistance (MIC = 512 mg/L). This shift in resistance is associated with increased expression of *bla*_NDM-5_. Increased MIC attributed to elevated expression and copy number of resistance genes is commonly reported in Gram-negative bacteria. For example, the overexpression of *bla*_NDM-1_ in *Klebsiella pneumoniae* leads to heightened resistance to carbapenem antibiotics (Ding et al., 2024). In addition, exposure to varying concentrations of ciprofloxacin results in increased expression of *qnrB1*, mediating resistance to fluoroquinolones (Gulyás et al., 2019).

The overexpression of efflux pumps is one of the factors contributing to resistance against carbapenem antibiotics in bacteria such as *E. coli*, *Salmonella*, *Pseudomonas aeruginosa*, and *Klebsiella pneumoniae* (Li et al., 2015; Lucaßen et al., 2021; Nishino et al., 2021; Zwama and Nishino, 2021). In *E. coli*, the RND-type efflux pump AcrAB-TolC system is closely associated with antibiotic resistance, mediating resistance to *β*-lactams, tetracyclines, macrolides, chloramphenicol, and quinolone antibiotics (Tam et al., 2021). Pump inhibition experiments have shown that efflux pumps contribute to changes in antibiotic resistance in various strains, with the AcrAB-TolC system being the main type implicated. Supporting these findings, Chetri et al. observed an increase in the expression of the *bla*_NDM-1_, *acrA*, and *acrB* genes in *E. coli* under the pressure of carbapenem (Chetri et al., 2019). Furthermore, research has indicated that the upregulated expression of efflux pump-related genes detected through transcriptome sequencing may result from interactions between the NDM-5 protein and efflux pump-related proteins (Li L. et al., 2024).

## Conclusion

In this study, we examined 103 *bla*_NDM-5_-positive, multiantibiotic-resistant *E. coli* strains isolated from 431 intestinal fecal samples of waterfowl across Sichuan, Anhui, Shaanxi, Xinjiang and Chongqing. Our experiments confirmed that the *bla*_NDM-5_ gene can be horizontally transferred via highly mobile plasmids, which, although exerting a fitness cost on host bacteria, have a minimal impact overall. One strain, DY51, exhibited a significant shift from low-level (MIC = 4 mg/L) resistance to high-level resistance (MIC = 512 mg/L) following *in vitro* induction, which was linked to increased *bla*_NDM-5_ expression and efflux pump activity.

This study raises significant public health concerns regarding AMR, particularly the presence of *bla*_NDM-5_-positive *E. coli* in waterfowl, which suggests that waterfowl may serve as reservoirs for AMR pathogens. Enhanced agricultural surveillance is essential. Future research should focus on environmental practices that contribute to the spread of AMR genes among waterfowl and the stability of resistance. Targeted interventions and monitoring for AMR in waterfowl are crucial for protecting human health and informing public health policies.

## Data Availability

The original contributions presented in the study are publicly available. This data can be found here: NCBI accession numbers: PQ621134–PQ621236.

## Footnotes

^1^http://www.ncbi.nlm.nih.gov/BLAST/
