# Supplementary material for: Decoding the enigma: unveiling the transmission characteristics of waterfowl-associated blaNDM-5-positive Escherichia coli in select regions of China
Source: Front Microbiol. 2024 Dec 9;15:1501594. doi: 10.3389/fmicb.2024.1501594 (PMC11663885; doi:10.3389/fmicb.2024.1501594)
Supplement: Supplementary file 1 [file Data_Sheet_1.docx]

**Appendix. Supplementary materials**

**Figures**

**
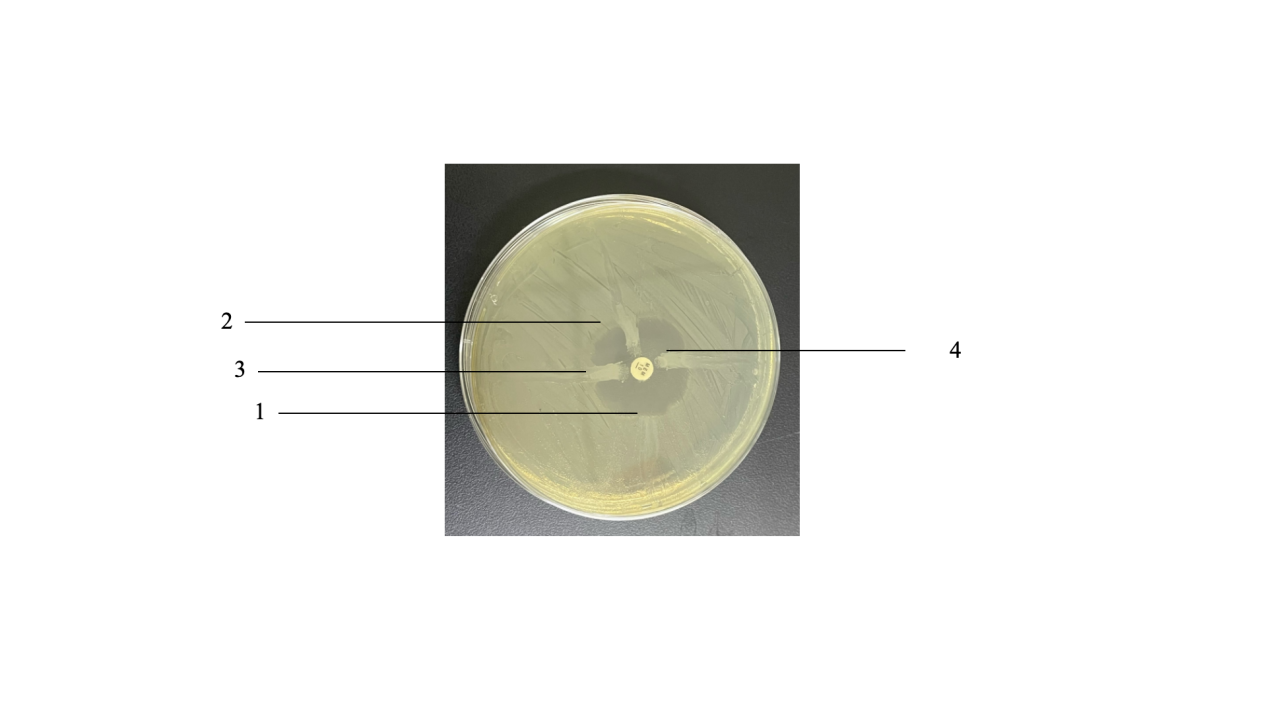
**

**Figure S1 Results of modified Hodge test**

Note：1: *E. coli* ATCC 25922, 2 , 3 and 4 are *bla*NDM-5 positive *E. coli*

**
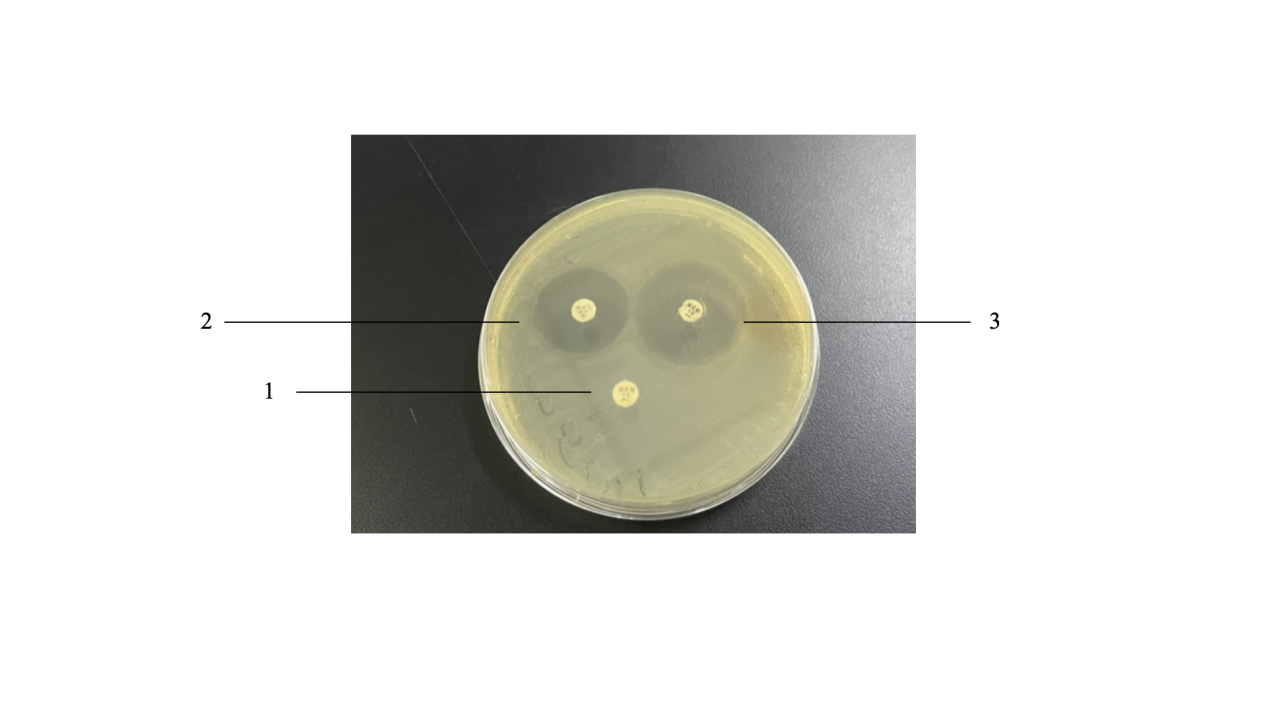
**

**Figure S2 Results of mCIM and eCIM test**

Note: 1: The *bla*NDM-5 gene positive *E. coli* mCIM was positive, and the inhibition zone diameter was 6 mm, 2: The *bla*NDM-5 gene positive *E. coli* eCIM was positive, and the inhibition zone diameter was 26 mm, 3: Negative control, *E. coli* ATCC 25922


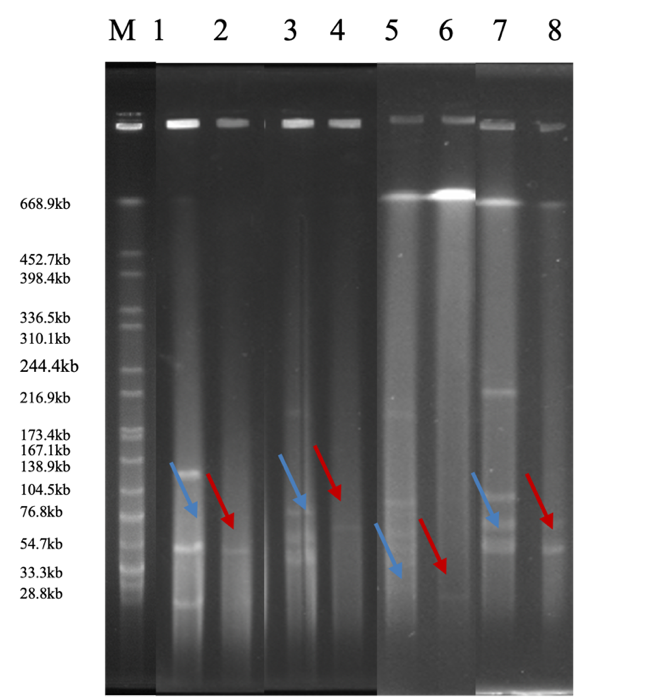


Figure S3 S1-PFGE results of 4 donor bacteria and 4 transconjugants

Note: M: *Salmonella* H9812 Marker ; lane 1: DY51, lane 2: DY51-J, lane 3: DY3-2, lane 4: DY3-2-J, lane 5: dy8-3, lane 6: dy8-3-J, lane 7: MS33-1, lane 8: MS33-1-J


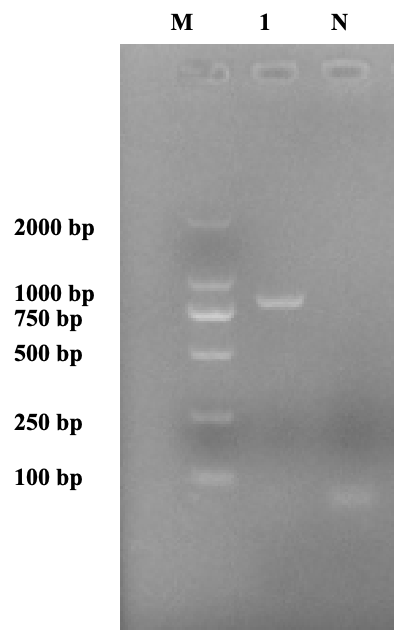


Figure S4 Identification of *bla*_NDM-5_ gene connection vector pET32a(+)

Note：M：DL2000 Marker，Lane 1 is a single colony of transformants grown after ligation transformation, N：negative control


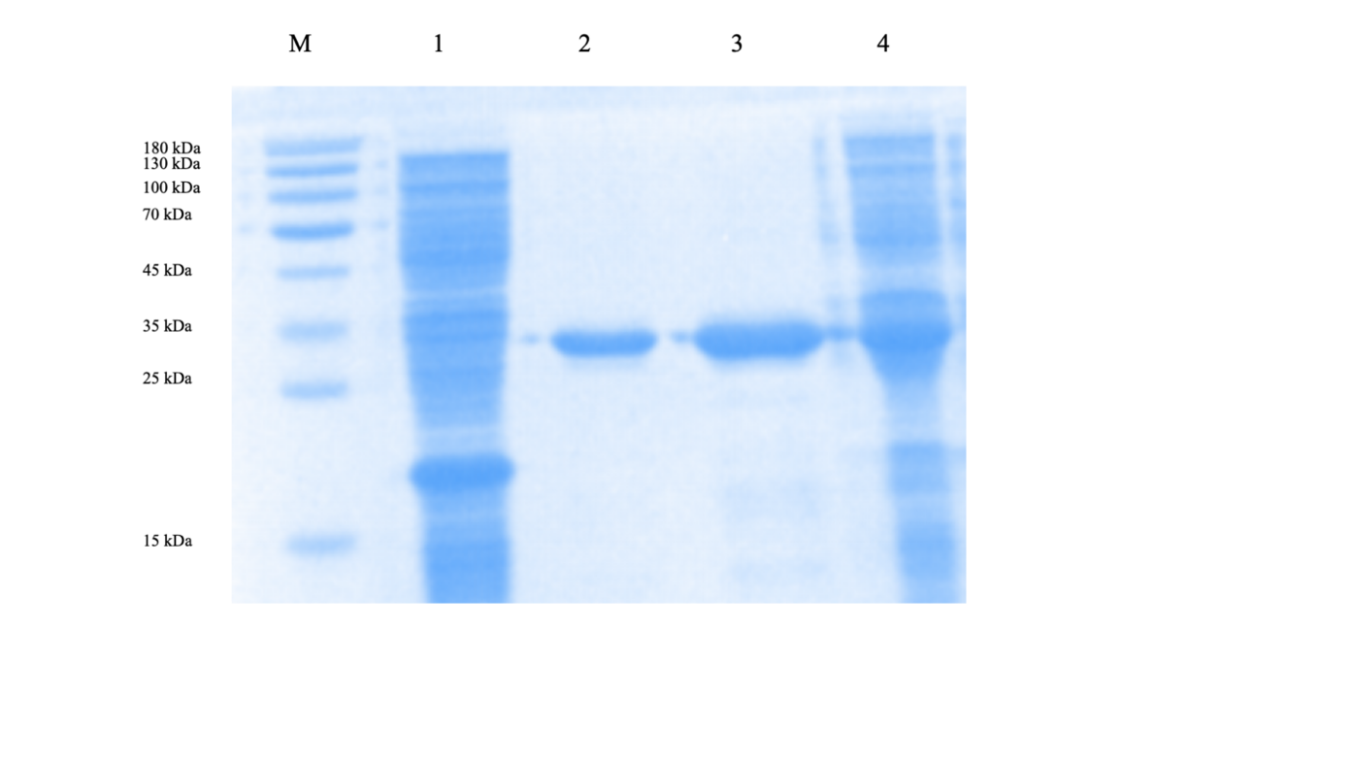


Figure S5 The purification of NDM-5 protein in *E. coli* BL21(DE3)-pET32a(+)-*bla*_NDM-5_ was detected by SDS-PAGE

Note : M：the protein marker, lane 1 is *E. coli* BL21(DE3)-pET32a(+), lane 2 and lane 3 are the purification situations in the purification process of NDM-5 protein ,lane 4 is the total protein of *E. coli* BL21(DE3)-pET32a(+)-*bla*_NDM-5_ after induction


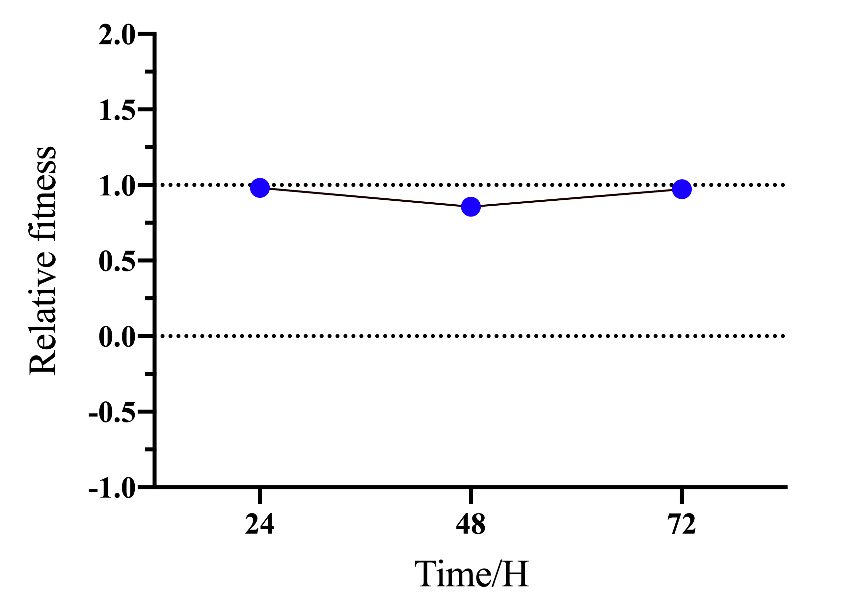


Figure S6 Relative adaptability of *bla*_NDM-5_ plasmid transformant


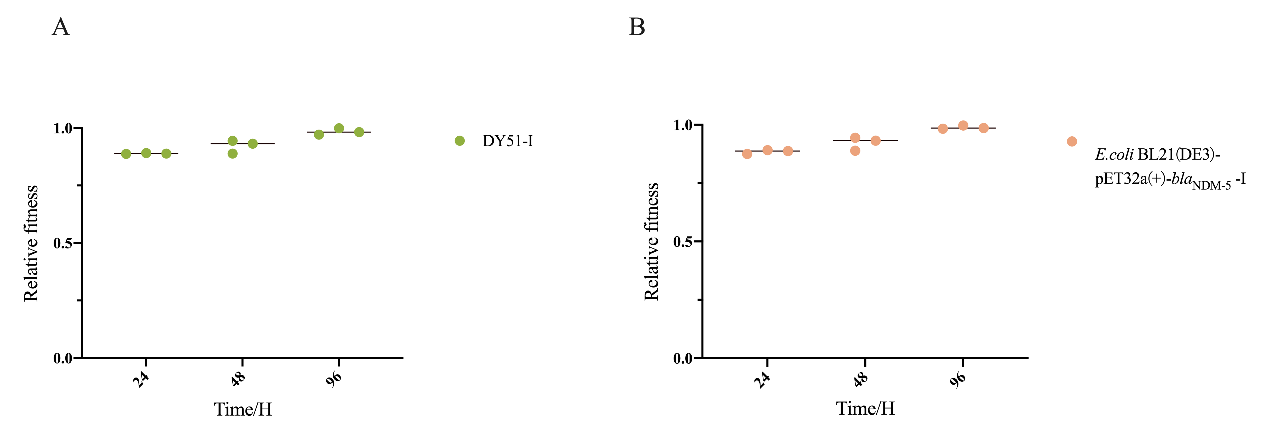


Figure S10 In vitro competition results of strains before and after induction

Note: Figure A shows the competition results of DY51 and induced strain DY51-I in vitro. Figure B shows the competition results of *E. coli* DH5α-pET32a(+)-*bla*_NDM-5_ and induced strain *E. coli* DH5α-pET32a(+)-*bla*_NDM-5_-I

**Tables**

**Table S1 Primer sequence of antibiotic resistance gene**

| Primer | Sequence（5’ – 3’） | Fragment Length  （bp） | Reference |
| --- | --- | --- | --- |
| *qnrA*-F | ATTTCTCACGCCAGGATTTG | 516 | (Robicsek et al., 2006) |
| *qnrA*-R | GATCGGCAAAGGTTAGGTCA |  |  |
| *qnrB*-F | GATCGTGAAAGCCAGAAAGG | 469 | (Wang et al., 2017) |
| *qnrB*-R | GATCGTGAAAGCCAGAAAGG |  |  |
| *qnrS*-F | ACGACATTCGTCAACTGCAA | 417 | (Wang et al., 2017) |
| *qnrS*-R | TAAATTGGCACCCTGTAGGC |  |  |
| *bla*_TEM_-F | GCGGAACCCCTATTTG | 964 | (Olesen et al., 2004) |
| *bla*_TEM_-R | ACCAATGCTTAATCAGTGAG |  |  |
| *bla*_NDM_-F | ATGGAATTGCCCAATATTATGCAC | 813 | (Hornsey et al., 2011) |
| *bla*_NDM_-R | TCAGCGCAGCTTGTCGGC |  |  |
| *bla*_SHV_-F | TTATCTCCCTGTTAGCCACC | 795 | (Ahmed et al., 2006) |
| *bla*_SHV_-R | GATTTGCTGATTTCGCTCGG |  |  |
| *bla*_CTX-M_-F | CGATGTGCAGTACCAGTAA | 585 | (Batchelor et al., 2005) |
| *bla*_CTX-M_*-*R | TAGTGACCAGAATCAGCGG |  |  |
| *aac（6’）-Ib-cr*-F | TTGCGATGCTCTATGAGTGGCTA | 482 | (Eftekhar and Seyedpour, 2015) |
| *aac（6’）-Ib-cr*-R | CTCGAATGCCTGGCGTGTTT |  |  |
| *aadA1*-F | TATCAGAGGTAGTTGGCGTCAT | 489 | (Niu et al., 2020) |
| *aadA1*-R | GCGAGTTCCATAGCGTTAAGG |  |  |
| *aac(3)-I*-F | ACCTACTCCCAACATCAGCC | 169 | (Sáenz et al., 2004) |
| *aac(3)-I*-R | ATATAGATCTCACTACGCGC |  |  |
| *sul1*-F | GTGACGGTGTTCGGCATTCT | 779 | (Boerlin et al., 2005) |
| *sul1*-R | CCGAGAAGGTGATTGCGCT |  |  |
| *bla*_NDM-5_-F | ATGGAATTGCCCAATATTATGCAC | 813 | (Hornsey et al., 2011) |
| *bla*_NDM-5_-R | TCAGCGCAGCTTGTCGGC |  |  |
| *sul2*-F | CGGCATCGTCAACATAACC | 722 | (Boerlin et al., 2005) |
| *sul2*-R | GTGTGCGGATGAAGTCAG |  |  |
| *sul3*-F | CATTCTAGAAAACAGTCGTAGTTCG | 990 | (Ramos et al., 2013) |
| *sul3-*R | CATCTGCAGCTAACCTAGGGCTTTGGA |  |  |
| *tet*A-F | GTAATTCTGAGCACTGTCGC | 937 | (Guardabassi et al., 2000) |
| *tet*A-R | CTGCCTGGACAACATTGCTT |  |  |
| *tet*B-F | CTCAGTATTCCAAGCCTTTG | 416 | (Guardabassi et al., 2000) |

**Table S1 Primer sequence of antibiotic resistance gene（Continued Table）**

| Primer | Sequence（5’ – 3’） | Fragment Length  （bp） | Reference |
| --- | --- | --- | --- |
| *tet*B-R | CTAAGCACTTGTCTCCTGTT |  |  |
| *tet*C-F | TCTAACAATGCGCTCATCGT | 570 | (Guardabassi et al., 2000) |
| *tet*C-R | GGTTGAAGGCTCTCAAGGGC |  |  |
| *tet*M-F | AGTGGAGCGATTACAGAA | 158 | (Adefisoye and Okoh, 2016) |
| *tet*M-R | CATATGTCCTGGCGTGTCTA |  |  |
| *floR*-F | GTCGAGAAATCCCATGAGTTCA | 1645 | (Cloeckaert et al., 2000) |
| *floR*-R | CAGACAGGATACCGACATTCAC |  |  |
| *cat1*-F | AACCAGACCGTTCAGCTGGAT | 550 | (Adefisoye and Okoh, 2016) |
| *cat1*-R | CCTGCCACTCATCGCAGTAC |  |  |
| *ermA/TR*-F | TCAGGAAAAGGACATTTTACC | 432 | (Sutcliffe et al., 1996) |
| *ermA/TR*-R | ATACTTTTTGTAGTCCTTCTT |  |  |
| *ermB*-F | GATACCGTTTACGAAATTGG | 364 | (Zhang et al., 2016) |
| *ermB*-R | GAATCGAGACTTGAGTGTGC |  |  |
| *mcr-1*-F | AGTCCGTTTGTTCTTGTGGC | 320 | (Rebelo et al., 2018) |
| *mcr-1*-R | AGATCCTTGGTCTCGGCTTG |  |  |

Table S2 103 strains of *bla*_NDM-5_-positive *E. coli* mCIM and eCIM results

| Name of strain | mCIM  (mm) | eCIM  (mm) | *E. coli* ATCC  25922 (mm) | Name of strain | mCIM  (mm) | eCIM  (mm) | *E. coli* ATCC 25922(mm) |
| --- | --- | --- | --- | --- | --- | --- | --- |
| DY3-2 | 6 | 24 | 32 | SX28-1 | 6 | 27 | 36 |
| DY3-3 | 6 | 25 | 33 | SX28-2 | 6 | 26 | 37 |
| DY3-8 | 6 | 24 | 32 | SX28-3 | 6 | 29 | 36 |
| DY4-2 | 6 | 25 | 31 | SX28-4 | 6 | 28 | 35 |
| DY4-7 | 6 | 28 | 33 | SX34-2 | 6 | 28 | 37 |
| dy8-3 | 6 | 24 | 32 | SX28-5 | 6 | 29 | 36 |
| DY16-10 | 6 | 26 | 31 | SX28-6 | 6 | 25 | 33 |
| SN18-2 | 6 | 23 | 29 | SX34-3 | 6 | 26 | 36 |
| MS19-2 | 6 | 26 | 33 | SX34-4 | 6 | 28 | 45 |
| dy21-5 | 6 | 23 | 30 | SX34-5 | 6 | 26 | 33 |
| dy21-6 | 6 | 26 | 34 | SX34-6 | 6 | 25 | 33 |
| XJ31-1 | 6 | 23 | 33 | SX34-7 | 6 | 27 | 32 |
| XJ31-2 | 6 | 24 | 33 | DY35-2 | 6 | 22 | 28 |
| XJ31-3 | 6 | 30 | 36 | DY35-1 | 6 | 29 | 36 |
| MS33-1 | 6 | 29 | 34 | DY35-5 | 6 | 27 | 36 |
| MS33-2 | 6 | 28 | 33 | DY35-7 | 6 | 28 | 35 |
| XJ22-1 | 6 | 25 | 34 | DY35-8 | 6 | 24 | 35 |
| XJ22-2 | 6 | 24 | 31 | DY35-3 | 6 | 25 | 33 |
| XJ22-3 | 6 | 27 | 35 | DY35-9 | 6 | 28 | 35 |
| XJ22-4 | 6 | 28 | 33 | DY35-10 | 6 | 23 | 31 |
| XJ22-5 | 6 | 27 | 31 | DY35-11 | 6 | 29 | 36 |
| XJ22-6 | 6 | 26 | 32 | DY35-12 | 6 | 26 | 33 |
| XJ23-2 | 6 | 29 | 36 | DY35-4 | 6 | 25 | 31 |
| XJ23-4 | 6 | 27 | 33 | DY35-13 | 6 | 25 | 34 |
| XJ23-6 | 6 | 27 | 33 | DY35-14 | 6 | 24 | 32 |
| XJ31-4 | 6 | 26 | 32 | DY35-15 | 6 | 23 | 29 |
| XJ31-5 | 6 | 27 | 34 | DY35-16 | 6 | 27 | 34 |
| XJ31-6 | 6 | 27 | 33 | DY35-6 | 6 | 26 | 32 |
| XJ31-7 | 6 | 28 | 33 | DY35-17 | 6 | 25 | 33 |
| XJ31-8 | 6 | 26 | 32 | DY35-18 | 6 | 25 | 31 |
| XJ31-9 | 6 | 26 | 32 | CZ37-1 | 6 | 27 | 37 |
| MS20-1 | 6 | 27 | 33 | CZ37-2 | 6 | 27 | 35 |
| MS20-2 | 6 | 26 | 34 | CZ37-3 | 6 | 25 | 33 |
| MS20-4 | 6 | 27 | 33 | CZ37-6 | 6 | 28 | 35 |
| MS25-1 | 6 | 25 | 31 | CZ37-7 | 6 | 27 | 33 |
| MS25-2 | 6 | 26 | 34 | CZ37-4 | 6 | 24 | 31 |
| SX34-1 | 6 | 25 | 33 | CZ37-8 | 6 | 26 | 32 |

Table S2 103 strains of *bla*_NDM-5_-positive *E. coli* mCIM and eCIM results（Continued Table）

| Name of strain | mCIM  (mm) | eCIM  (mm) | *E. coli* ATCC  25922 (mm) | Name of strain | mCIM  (mm) | eCIM  (mm) | *E. coli* ATCC 25922(mm) |
| --- | --- | --- | --- | --- | --- | --- | --- |
| CZ37-10 | 6 | 25 | 32 | CZ37-9 | 6 | 28 | 33 |
| CZ37-11 | 6 | 28 | 35 | MS38-1 | 6 | 25 | 31 |
| CZ37-5 | 6 | 25 | 33 | MS38-2 | 6 | 26 | 33 |
| CZ37-12 | 6 | 26 | 35 | MS38-4 | 6 | 28 | 34 |
| CZ37-13 | 6 | 26 | 33 | MS38-5 | 6 | 27 | 35 |
| CZ37-14 | 6 | 24 | 31 | JT39-1 | 6 | 25 | 33 |
| CZ37-15 | 6 | 29 | 34 | JT39-2 | 6 | 26 | 35 |
| SN42-4 | 6 | 27 | 33 | SN42-1 | 6 | 25 | 31 |
| LS44-4 | 6 | 26 | 32 | SN42-3 | 6 | 29 | 35 |
| LS44-5 | 6 | 24 | 31 | SN42-5 | 6 | 29 | 34 |
| LS44-6 | 6 | 28 | 33 | SN42-6 | 6 | 27 | 34 |
| DY51 | 6 | 27 | 33 | SN42-7 | 6 | 25 | 31 |
| DY43-2 | 6 | 28 | 33 | SN42-10 | 6 | 27 | 33 |
| DY43-6 | 6 | 24 | 32 | DY43-1 | 6 | 27 | 31 |
| LS44-3 | 6 | 24 | 31 |  |  |  |  |

**Table S3 Correlation analysis of drug resistance genes in 103 strains of *bla*_NDM-5_-positive *Escherichia coli* from waterfowl**

| Gene | OR (95% confidence interval) | | | | | | | | |
| --- | --- | --- | --- | --- | --- | --- | --- | --- | --- |
|  | *qnrS* | *bla*_TEM_ | *bla*_SHV_ | *bla*_CTX-M_ | *bla*_KPC_ | *aac(6’)-Ibcr* | *aadA1* | *aac(3)-I* | *sul1* |
| *qnrS* | NS | - | - | - | - | 0.092  （0.012-0.723） | 4.721  （1.497-14.889） | - | - |
| *bla*_TEM_ | - | NS | 0.875  （0.793-0.96） | 0.153  （0.0.30-0.785） | - | - | - | - | - |
| *bla*_SHV_ | - | 0.505  （0.415-0.615） | NS | - | - | - | - | - | - |
| *bla*_CTX-M_ | - | 0.153  （0.030-0.785） | - | NS | - | - | 4.467  （1.331-14.990） | - | - |
| *bla*_KPC_ | - | - | - | - | NS | - | - | - | - |
| *aac(6’)-Ibcr* | 0.092  （0.012-0.723） | - | - | - | - | NS | - | - | 1.487  （1.243-1.780） |
| *aadA1* | 4.721  （1.497-14.889） | - | - | 4.467  （1.331-14.990） | - | - | NS | - | - |
| *aac(3)-I* | - | - | - | - | - | - | - | NS | - |
| *sul1* | *-* | - | - | - | - | 0.459  （0.364-0.578） | - | - | NS |
| *sul2* | 0.290  （0.106-0.794） | - | - | - | - | 0.765  （0.680-0.860） | - | - | 18.000  （3.899-83.101） |
| *sul3* | - | 1.400  （0.876-2.237） | - | - | - | - | - | - | - |
| *tet*A | - | - | - | 6.234  (1.769-21.965) | - | - | 4.221  （1.433-12.430） | - | - |
| *tet*B | - | 1.400  （0.876-2.237） | - | 0.846  （0.671-1.067） | - | - | - | - | - |
| *tet*C | - | - | 6.231  （1.275-30.449） | - | - | - | 1.180  （1.070-1.302） | - | 0.063  （0.008-0.514） |
| *floR* | - | - | - | - | - | - | - | - | - |
| *cat1* | - | 9.643  （1.792-51.892） | - | - | - | 30.182（5.669-160.692） | - | - | 8.265（1.007-67.851） |
| *ermB* | - | 16.00  (0.888-288.445) | - | - | - | 1.118  （0.958-1.304） | - | - | - |
| *mcr-1* | - | - | - | 1.422  （1.244-1.625） | - | - | 3.286  （1.031-10.469） | - | - |
| *qnrS* | 0.290  （0.106-0.794） | - | - | - | - | - | - | - | - |

**Table S3 Correlation analysis of drug resistance genes in 103 strains of *bla*_NDM-5_-positive *Escherichia coli* from waterfowl（Continued Table）**

| Gne | OR（95% confidence interval） | | | | | | | | |
| --- | --- | --- | --- | --- | --- | --- | --- | --- | --- |
|  | *sul2* | *sul3* | *tet*A | *tet*B | *tet*C | *floR* | *cat1* | *ermB* | *mcr-1* |
| *bla*_TEM_ | - | 0.049  （0.021-0.121） | - | 0.049  （0.021-0.121） | - | - | 9.643  （1.792-51.892） | 16.00  （0.888-880.445） | - |
| *bla_S_*_HV_ | - | - | - | - | 6.231  （1.275-30.449） | - | - | - | - |
| *bla*_CTX-M_ | - | - | 6.234  （1.769-1.965） | 0.108  （0.062-0.188） | - | - | - | - | 0.831  （0.752-0.919） |
| *bla*_KPC_ | - | - | - | - | - | - | - | - | - |
| *aac(6’)-Ib-cr* | 1.292  （1.151-1.451） | - | - | - | - | - | 30.182  （5.669-160.692） | 0.167  （0.108-0.257） | - |
| *aadA1* | - | - | 4.221  （1.433-12.430） | - | 0.656  （0.566-0.760） | - | - | - | 3.286  （1.031-10.469） |
| *aac(3)-I* | - | - | - | - | - | - | - | - | - |
| *sul1* | 18.000  （3.899-83.101） | - | - | - | 0.063  （0.008-0.514） | - | 8.265  （1.007-67.851） | - | - |
| *sul2* | NS | - | - | - | 0.030  （0.006-0.156） | 4.714  （1.378-16.131） | - | - | - |
| *sul3* | - | NS | - | 101.000  （3.370-3027.447） | - | - | 10.333  （0.595-179.537） | - | - |
| *tet*A | - | - | NS | - | - | 4.115  （1.154-14.676） | - | - | - |
| *tet*B | - | 101．0  （3.370-3027.447） | - | NS | - | - | 1.250  （0.917-1.704） | 101.000  （3.370-3027.447） | - |
| *tet*C | 0.030  （0.006-0.156） | - | - | - | NS | - | - | - | 18.750  （3.725-94.389） |
| *floR* | 4.714  （1.378-16.131） | - | 4.115  （1.154-14.676） | - | - | NS | - | - | - |
| *cat1* | - | 10.333  （0.595-179.537） | - | 0.078  （0.040-0.153） | - | - | NS | 10.333  （0.595-179.537） | 0.870  （0.798-0.949） |
| *ermB* | - | - | - | 101.000  （3.370-3027.447） | - | - | 10.333  （0.595-179.537） | NS | - |
| *mcr-1* | - | - | - | - | 18.750  （3.725-94.389） | - | 0.713  （0.627-0.810） | - | NS |

**Table S4 MICs of 103 *bla*_NDM-5_ positive *E. coli* strains to imipenem were determined**

| Name of strain | MIC（mg/L） | Name of strain | MIC（mg/L） | Name of strain | MIC（mg/L） |
| --- | --- | --- | --- | --- | --- |
| DY3-1 | 64 | MS25-2 | 512 | CZ37-4 | 512 |
| DY3-2 | 256 | SX34-1 | 512 | CZ37-5 | 512 |
| DY3-3 | 256 | SX28-1 | 512 | CZ37-6 | 512 |
| DY3-8 | 256 | SX28-2 | 512 | CZ37-7 | 256 |
| DY4-2 | 256 | SX28-3 | 512 | CZ37-8 | 256 |
| DY4-7 | 256 | SX28-4 | 512 | CZ37-9 | 512 |
| dy8-3 | 256 | SX34-2 | 512 | CZ37-10 | 512 |
| DY16-10 | 512 | SX28-5 | 256 | CZ37-11 | 512 |
| SN18-2 | 256 | SX28-6 | 512 | CZ37-12 | 64 |
| MS19-2 | 64 | SX34-3 | 512 | CZ37-13 | 512 |
| dy21-5 | 256 | SX34-4 | 512 | CZ37-14 | 512 |
| dy21-6 | 64 | SX34-5 | 512 | CZ37-15 | 256 |
| XJ31-1 | 512 | SX34-6 | 256 | MS38-1 | 128 |
| XJ31-2 | 256 | SX34-7 | ≥512 | MS38-2 | 128 |
| XJ31-3 | 512 | DY35-1 | ≥512 | MS38-4 | 256 |
| MS33-1 | 256 | DY35-2 | ≥512 | MS38-5 | 128 |
| MS33-2 | 256 | DY35-3 | 64 | JT39-1 | 128 |
| XJ22-1 | ≥512 | DY35-4 | 256 | JT39-2 | 64 |
| XJ22-2 | 512 | DY35-5 | ≥512 | SN42-1 | ≥512 |
| XJ22-3 | 512 | DY35-6 | 512 | SN42-3 | ≥512 |
| XJ22-4 | ≥512 | DY35-7 | 512 | SN42-4 | ≥512 |
| XJ22-5 | ≥512 | DY35-8 | 128 | SN42-5 | ≥512 |
| XJ22-6 | 512 | DY35-9 | 64 | SN42-6 | ≥512 |
| XJ23-2 | 512 | DY35-10 | 256 | SN42-7 | ≥512 |
| XJ23-4 | 512 | DY35-11 | 128 | SN42-10 | ≥512 |
| XJ23-6 | 512 | DY35-12 | 64 | DY43-1 | ≥512 |
| XJ31-4 | 512 | DY35-13 | 64 | DY43-2 | 64 |
| XJ31-5 | 512 | DY35-14 | 64 | DY43-6 | ≥512 |
| XJ31-6 | 512 | DY35-15 | 256 | LS44-3 | 64 |
| XJ31-7 | ≥512 | DY35-16 | 256 | LS44-4 | ≥512 |
| XJ31-8 | ≥512 | DY35-17 | 256 | LS44-5 | 64 |
| XJ31-9 | 512 | DY35-18 | 512 | LS44-6 | ≥512 |
| MS20-1 | ≥512 | CZ37-1 | 512 | DY51 | 4 |
| MS20-4 | 512 | CZ37-2 | 128 |  |  |
| MS25-1 | ≥512 | CZ37-3 | 256 |  |  |

**Table S5 The results of correlation analysis between antibiotic resistance genes and AMR phenotypes**

|  | OR（95% confidence interval） | | | | | | | | | | | | | | | | | |
| --- | --- | --- | --- | --- | --- | --- | --- | --- | --- | --- | --- | --- | --- | --- | --- | --- | --- | --- |
|  | *qnrS* | *bla*_TEM_ | *bla*_SHV_ | *bla*_CTX-M_ | *bla*_KPC_ | *aac(6’)-Ibcr* | *aadA1* | *aac(3)-I* | *sul1* | *sul2* | *sul3* | *tet*A | *tet*B | *tet*C | *floR* | *cat1* | *ermB* | *mcr-1* |
| CTX | - | - | - | - | - | - | - | - | - | - | - | - | - | - | - | - | - | - |
| TE | - | - | - | - | - | - | 0.813  （0.688-0.960） | - | - | - | - | 6.000  （1.099-32.758） | - | - | - | - | - | - |
| C | 0.845  （0.765-0.934） | - | - | - | - | - | 7.667（1.880-31.272） | - | - | - | - | 5.625（1.484 -21.326） | - | - | - | - | - | - |
| AMP | NA | NA | NA | NA | NA | NA | NA | NA | NA | NA | NA | NA | NA | NA | NA | NA | NA | NA |
| PB | - | - | - | - | - | 0.812（0.733-0.899） | 0.275（0.092-0.824） | - | - | - | - | - | - | 3.84  （0.980-15.178） | - | - | - | - |
| NFX | - | - | - | - | - | 0.101（0.009-1.180） | - | - | - | - | - | - | - | - | - | - | 0.020（0.001-0.446） | - |
| ATM | - | - | - | - | - | - | - | - | -- | - | - | 4.836（1.525- 15.342） | - | - | - | - | - | - |
| KAN | - | - | - | - | - | - | 13.696（2.757-68.036） | - | - | - | - | - | - | - | - | - | - | - |
| AZM | 0.270  （0.085-0.858） | - | - | - | - | - | 0.159（0.062-0.410） | - | - | - | - | - | - | - | - | - | 0.255（0.183-0.355） | 0.260（0.071-0.946） |
| SXT | - | - | - | - | - | - | 1.180（1.070-1.302） | - | - | - | - | - | - | - | - | - | - | - |

**Reference**

Adefisoye, M.A., and Okoh, A.I. (2016). Identification and antimicrobial resistance prevalence of pathogenic *Escherichia coli* strains from treated wastewater effluents in Eastern Cape, South Africa. *MicrobiologyOpen* 5**,** 143-151.

Ahmed, A.M., Furuta, K., Shimomura, K., Kasama, Y., and Shimamoto, T. (2006). Genetic characterization of multidrug resistance in *Shigella spp.* from Japan. *Journal of Medical Microbiology* 55**,** 1685-1691.

Batchelor, M., Hopkins, K., Threlfall, E.J., Clifton-Hadley, F.A., Stallwood, A.D., Davies, R.H., and Liebana, E. (2005). *bla*_CTX-M_ genes in clinical *Salmonella* isolates recovered from humans in England and Wales from 1992 to 2003. *Antimicrobial Agents and Chemotherapy* 49**,** 1319-1322.

Boerlin, P., Travis, R., Gyles, C.L., Reid-Smith, R., Janecko, N., Lim, H., Nicholson, V., Mcewen, S.A., Friendship, R., and Archambault, M. (2005). Antimicrobial resistance and virulence genes of *Escherichia coli* isolates from swine in Ontario. *Applied and Environmental Microbiology* 71**,** 6753-6761.

Cloeckaert, A., Baucheron, S., Flaujac, G., Schwarz, S., Kehrenberg, C., Martel, J.L., and Chaslus-Dancla, E. (2000). Plasmid-mediated florfenicol resistance encoded by the *floR* gene in *Escherichia coli* isolated from cattle. *Antimicrobial Agents and Chemotherapy* 44**,** 2858-2860.

Eftekhar, F., and Seyedpour, S.M. (2015). Prevalence of *qnr* and *aac(6')-Ib-cr* Genes in Clinical Isolates of *Klebsiella Pneumoniae* from Imam Hussein Hospital in Tehran. *Iranian Journal of Medical Sciences* 40**,** 515-521.

Guardabassi, L., Dijkshoorn, L., Collard, J.M., Olsen, J.E., and Dalsgaard, A. (2000). Distribution and in-vitro transfer of tetracycline resistance determinants in clinical and aquatic *Acinetobacter* strains. *Journal of Medical Microbiology* 49**,** 929-936.

Hornsey, M., Phee, L., and Wareham, D.W. (2011). A novel variant, NDM-5, of the New Delhi metallo-β-lactamase in a multidrug-resistant *Escherichia coli* ST648 isolate recovered from a patient in the United Kingdom. *Antimicrobial Agents and Chemotherapy* 55**,** 5952-5954.

Niu, J.-L., Peng, J.-J., Ming, Y.-Y., Ma, Q.-C., Liu, W.-C., and Ma, Y. (2020). Identification of drug resistance genes and drug resistance analysis of *Salmonella* in the duck farm environment of Zhanjiang, China. *Environmental Science and Pollution Research International* 27**,** 24999-25008.

Olesen, I., Hasman, H., and Aarestrup, F.M. (2004). Prevalence of beta-lactamases among ampicillin-resistant *Escherichia coli* and *Salmonella* isolated from food animals in Denmark. *Microbial Drug Resistance* 10**,** 334-340.

Ramos, S., Silva, N., Caniça, M., Capelo-Martinez, J.L., Brito, F., Igrejas, G., and Poeta, P. (2013). High prevalence of antimicrobial-resistant *Escherichia coli* from animals at slaughter: a food safety risk. *Journal of the Science of Food and Agriculture* 93**,** 517-526.

Rebelo, A.R., Bortolaia, V., Kjeldgaard, J.S., Pedersen, S.K., Leekitcharoenphon, P., Hansen, I.M., Guerra, B., Malorny, B., Borowiak, M., Hammerl, J.A., Battisti, A., Franco, A., Alba, P., Perrin-Guyomard, A., Granier, S.A., De Frutos Escobar, C., Malhotra-Kumar, S., Villa, L., Carattoli, A., and Hendriksen, R.S. (2018). Multiplex PCR for detection of plasmid-mediated colistin resistance determinants, *mcr-1*, *mcr-2*, *mcr-3*, *mcr-4* and *mcr-5* for surveillance purposes. *Euro Surveillance* 23.

Robicsek, A., Strahilevitz, J., Sahm, D.F., Jacoby, G.A., and Hooper, D.C. (2006). *qnr* prevalence in ceftazidime-resistant *Enterobacteriaceae* isolates from the United States. *Antimicrobial Agents and Chemotherapy* 50**,** 2872-2874.

Sáenz, Y., Briñas, L., Domínguez, E., Ruiz, J., Zarazaga, M., Vila, J., and Torres, C. (2004). Mechanisms of resistance in multiple-antibiotic-resistant *Escherichia coli* strains of human, animal, and food origins. *Antimicrobial Agents and Chemotherapy* 48**,** 3996-4001.

Sutcliffe, J., Grebe, T., Tait-Kamradt, A., and Wondrack, L. (1996). Detection of erythromycin-resistant determinants by PCR. *Antimicrobial Agents and Chemotherapy* 40**,** 2562-2566.

Wang, Y., Zhang, A., Yang, Y., Lei, C., Jiang, W., Liu, B., Shi, H., Kong, L., Cheng, G., Zhang, X., Yang, X., and Wang, H. (2017). Emergence of *Salmonella enterica* serovar Indiana and California isolates with concurrent resistance to cefotaxime, amikacin and ciprofloxacin from chickens in China. *International Journal of Food Microbiology* 262**,** 23-30.

Zhang, J., Chen, M., Sui, Q., Wang, R., Tong, J., and Wei, Y. (2016). Fate of antibiotic resistance genes and its drivers during anaerobic co-digestion of food waste and sewage sludge based on microwave pretreatment. *Bioresource Technology* 217**,** 28-36.
